# Motor versus Psychomotor? Deciphering the Neural Source of Psychomotor Retardation in Depression

**DOI:** 10.1002/advs.202403063

**Published:** 2024-08-29

**Authors:** Xue Mei Song, Dong‐Yu Liu, Dusan Hirjak, Xi‐Wen Hu, Jin‐Fang Han, Anna Wang Roe, De‐Zhong Yao, Zhong‐Lin Tan, Georg Northoff

**Affiliations:** ^1^ Department of Neurosurgery of the Second Affiliated Hospital Interdisciplinary Institute of Neuroscience and Technology School of Medicine Zhejiang University Hangzhou 310029 China; ^2^ Key Laboratory of Biomedical Engineering of Ministry of Education Qiushi Academy for Advanced Studies College of Biomedical Engineering and Instrument Science Zhejiang University Hangzhou 310027 China; ^3^ Department of Psychiatry and Psychotherapy Central Institute of Mental Health Medical Faculty Mannheim University of Heidelberg 69117 Mannheim Germany; ^4^ Affiliated Mental Health Center & Hangzhou Seventh People's Hospital School of Medicine Zhejiang University Hangzhou 310013 China; ^5^ The Clinical Hospital of Chengdu Brain Science Institute MOE Key Laboratory for Neuroinformation University of Electronic Science and Technology of China Chengdu 610054 China; ^6^ University of Ottawa Institute of Mental Health Research University of Ottawa Ottawa ON K1Z 7K4 Canada

**Keywords:** major depressive disorder, middle temporal visual complex, motion perception, primary motor cortex, psychomotor retardation

## Abstract

Major depressive disorder (MDD) is characterized by psychomotor retardation whose underlying neural source remains unclear. Psychomotor retardation may either be related to a motor source like the motor cortex or, alternatively, to a psychomotor source with neural changes outside motor regions, like input regions such as visual cortex. These two alternative hypotheses in main (*n* = 41) and replication (*n* = 18) MDD samples using 7 Tesla MRI are investigated. Analyzing both global and local connectivity in primary motor cortex (BA4), motor network and middle temporal visual cortex complex (MT+), the main findings in MDD are: 1) Reduced local and global synchronization and increased local‐to‐global output in motor regions, which do not correlate with psychomotor retardation, though. 2) Reduced local‐to‐local BA4 – MT+ functional connectivity (FC) which correlates with psychomotor retardation. 3) Reduced global synchronization and increased local‐to‐global output in MT+ which relate to psychomotor retardation. 4) Reduced variability in the psychophysical measures of MT+ based motion perception which relates to psychomotor retardation. Together, it is shown that visual cortex MT+ and its relation to motor cortex play a key role in mediating psychomotor retardation. This supports psychomotor over motor hypothesis about the neural source of psychomotor retardation in MDD.

## Graphical abstract

Using 7 Tesla MRI and analyzing both global and local synchronization in motor and visual cortex, this work finds psychomotor retardation relates to visual cortex on both neural and perceptual levels which, through globally mediated synchronization, down modulates neural activity in motor cortex. This supports psychomotor over motor model of psychomotor retardation in depression which carries diagnostic and therapeutic implications.

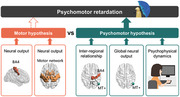

## 1 Introduction

Major depressive disorder (MDD) is a mood disorder that, besides its affective, somatic, and cognitive symptoms, also shows abnormal slowness in its movements, e.g., psychomotor retardation.^[^ ^1^, ^2^, ^3^ ^]^ While it has often been neglected in neuroscientific research,^[^ ^4^, ^5^ ^]^ psychomotor retardation is clinically of key importance as it precedes the onset of acute mood and cognitive symptoms,^[^ ^6^ ^]^ predicts depression severity^[^ ^7^ ^]^ and, conversely, recovers early as first sign of improvement.^[^ ^6^ ^]^ Therefore, there is an urgent need to delineate its underlying neural mechanisms to serve as biomarkers for both early warning sign of an upcoming depression and therapeutic marker of improvement.^[^ ^8^, ^9^ ^]^

Different models of the neural source (understood in a rather broad noncausal way here) of psychomotor retardation in MDD have been suggested. The “motor” model of psychomotor retardation highlights aberrant local regional activity and connectivity within the primary motor cortex itself and its associated subcortical motor systems like the basal ganglia and the cerebellum.^[^ ^5^, ^10^, ^11^, ^12^ ^]^ In contrast, the “psychomotor” model of psychomotor retardation suggests that psychomotor retardation in MDD is related to globally mediated neural activity in nonmotor regions like frontal cortex,^[^ ^13^, ^14^, ^15^ ^]^ default mode network (DMN),^[^ ^16^ ^]^ hippocampus,^[^ ^17^ ^]^ and middle temporal visual cortex complex (MT+).^[^ ^18^, ^19^ ^]^

Earlier studies showed reduced GABA levels in visual cortex in MDD^[^ ^20^, ^21^, ^22^ ^]^ which are modulated by different kinds of treatment.^[^ ^8^, ^22^, ^23^ ^]^ The role of visual cortex and especially its MT+ in MDD could recently be further confirmed by showing globally mediated decreased activity,^[^ ^19^, ^24^ ^]^ relationship with early childhood traumatic life events,^[^ ^25^, ^26^ ^]^ and impaired visual psychophysical performance.^[^ ^18^, ^23^ ^]^ Most interestingly in the present context, abnormal neural activity in the resting state of visual cortex MT+ relates to psychomotor retardation.^[^ ^18^ ^]^ This is further supported by another study showing that psychomotor retardation relates to reduced activity in visual cortex and DMN regions during the stimulation with especially fast visual stimuli.^[^ ^16^ ^]^ Together, these findings suggest a key role of slower dynamics in visual cortex MT+ in mediating psychomotor retardation in MDD. While these findings are in line with the psychomotor rather than the motor source of psychomotor retardation, the exact pathways of the effect of visual cortex MT+ on psychomotor retardation remain yet unclear, though. Further, the psychomotor model is based on widespread and more or less global connectivity changes in various regions and networks in MDD beyond and outside the cortical and subcortical motor regions including other regions like anterior cingulate,^[^ ^27^ ^]^ DMN,^[^ ^21^, ^24^, ^28^ ^]^ and lateral prefrontal cortex.^[^ ^29^, ^30^ ^]^ These global changes also include reduced activity in primary sensory regions like the visual cortex^[^ ^19^, ^22^, ^24^, ^31^ ^]^ whose relation to the motor cortex and role in psychomotor retardation remains yet unclear, though.

**Figure 1:**
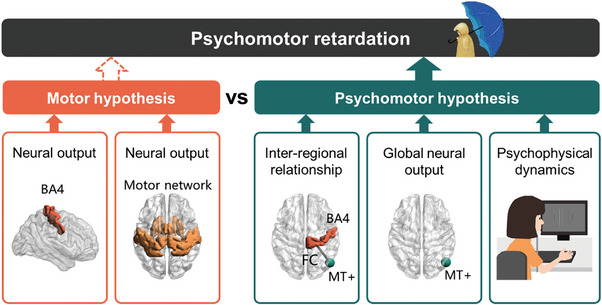
Motor and psychomotor hypotheses of the pathogenesis of psychomotor retardation in MDD. Motor and psychomotor models. BA, Brodmann Area; FC, functional connectivity; MT+, middle temporal visual cortex complex.

Based on this evidence, the main goal of this study is to examine the two alternative models about the neural source of psychomotor retardation in MDD, that is, motor and psychomotor model (**Figure** **1**). This study has three specific aims: The first aim is to investigate local regional and global synchronization as well as local‐to‐global output to the whole brain in motor regions, e.g., Brodmann Area 4 (BA4) and motor network themselves and relate them to psychomotor retardation. If the motor model holds, one would expect reduced local regional synchronization within BA4 and motor network themselves to correlate with psychomotor retardation. In contrast, in case of the psychomotor model, one would still expect altered representation of global synchronization and local‐to‐global output in BA4 and motor network although without any relationship with psychomotor retardation. The second aim consists in investigating the functional connectivity (FC) of BA4 and motor network with MT+ including its relation to psychomotor retardation. While BA4 − MT+ FC may be reduced in the motor model, it should not relate to psychomotor retardation, as this would indicate a neural source outside the motor cortex itself. In contrast, the psychomotor model would assume that BA4 − MT+ FC should be related to psychomotor retardation in, for instance, the case of primary visual cortex input changes and global cortical changes. The third aim is to investigate visual cortex MT+ itself in both its local and global synchronization as well as its local‐to‐global output to the whole brain. Further we investigated MT+ based perceptual dynamics in its psychophysical measures including their relation to psychomotor retardation. If the motor model holds, there should be no relationship of both MT+ based neural measures and perceptual dynamics with psychomotor retardation whereas the psychomotor model would be well compatible with such relation.

## 2 Results

### 2.1 Reduced Neural Synchronization in Primary Motor Cortex and Motor Network do not Relate to Psychomotor Retardation

**Figure 2:**
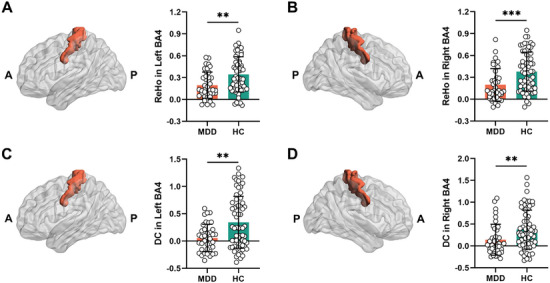
Group difference in ReHo and DC within BA4 between MDD and HC. MDD exhibited significantly reduced ReHo A,B) and DC C,D) in left and right BA4 compared with HC. **P_FDR_ < 0.01, ***P_FDR_ < 0.001, Student's *t* test. A, anterior; P, posterior; ReHo, regional homogeneity; DC, degree centrality; BA, Brodmann Area; MDD, major depressive disorder; HC, healthy control.

We first investigated local synchronization in primary motor cortex and motor network as indexed by regional homogeneity (ReHo). This was found to be decreased in both left BA4 (*T* = −3.35, *P_FDR_* = 0.001) (**Figure** **2**A), right BA4 (*T* = −3.62, *P_FDR_* < 0.001) (Figure 2B) and motor network (*T* = −3.56, *P* < 0.001) (Figure S1A, Supporting Information) in MDD. Next, we investigated the degree of global synchronization as indexed by degree centrality (DC). DC was significantly reduced in both left BA4 (*T* = −3.46, *P_FDR_* = 0.002) (Figure 2C), right BA4 (*T* = −2.81, *P_FDR_* = 0.006) (Figure 2D) and motor network (*T* = −2.55, *P* = 0.012) (Figure S1B, Supporting Information) in MDD.

Furthermore, DC positively correlated with ReHo in left BA4 (*R* = 0.536, *P* = 0.0006) (Figure S2A, Supporting Information), right BA4 (*R* = 0.803, *P* < 0.0001) (Figure S2B, Supporting Information), and motor network (*R* = 0.711, *P* < 0.001) (Figure S1C, Supporting Information) of MDD. Similar results were found in the healthy control (HC) group (Figures S1D and S2C,D, Supporting Information).

We next raised the question whether the reduced global and local neural synchronization in motor cortex relate to psychomotor retardation. No significant correlations with psychomotor retardation in all its three measures, e.g., the items score as well as their division by total Hamilton Depression Rating Scale (HAMD) with and without retardation, were obtained for both local and global neural measures of primary motor cortex synchronization (Figure S2E, Supporting Information). Neither was ReHo in motor network correlated with HAMD total score (*R* = 0.253, *P* = 0.111) and psychomotor retardation in all its three measures, e.g., the items score (*R* = 0.184, *P* = 0.248) as well as their division by total HAMD with (*R* = −0.028, *P* = 0.860) and without retardation (*R* = −0.077, *P* = 0.631). DC in motor network was also not correlated with HAMD total score (*R* = 0.115, *P* = 0.475) and psychomotor retardation score (*R* = −0.093, *P* = 0.562) as well as their division by total HAMD with (*R* = −0.209, *P* = 0.190) and without retardation (*R* = −0.222, *P* = 0.163). These results were similar to those in BA4. In sum, these findings show significant reduction in both global and local neural synchronization in the primary motor cortex and motor network of MDD. However, neither reduction in global nor in local synchronization of BA4 and motor network relate to psychomotor retardation.

### 2.2 Globally Mediated Decreased FC of BA4 with MT+ Relates to Psychomotor Retardation

**Figure 3:**
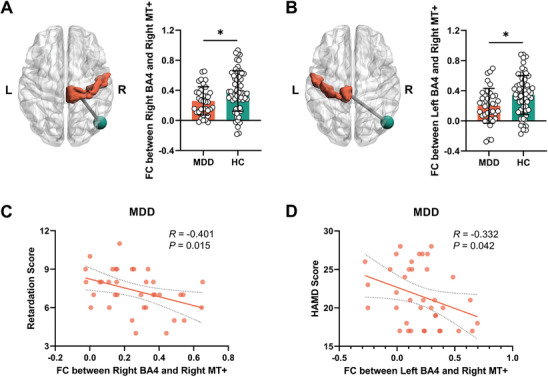
Comparison of the functional connectivity (FC) between BA4 and right MT+ between MDD and HC groups. FC between right A) and left BA4 B) and right MT+ were decreased in MDD group (Student's *t* test). C) The relationship between retardation score and FC between right BA4 and right MT+ in MDD group (Pearson correlation). D) The correlation between HAMD total score and FC between left BA4 and right MT+ in MDD group (Pearson correlation). *P_FDR_ < 0.05. L, left; R, right; FC, functional connectivity; BA, Brodmann Area; MT+, middle temporal visual cortex complex; MDD, major depressive disorder; HC, healthy control.

We next searched for the neural source of reduced primary motor cortex and motor network synchronization beyond the motor regions themselves and whether that relates to psychomotor retardation. Given recent findings in visual cortex regions like MT+ and their relation to other cortical regions like motor cortex and prefrontal cortex as well as to psychomotor retardation,^[^ ^8^, ^18^, ^19^, ^23^ ^]^ we investigated whether BA4 exhibits reduced synchronization with MT+ by measuring their globally mediated functional connectivity. Compared with HC group, globally mediated FC of both right (*T* = −2.59, *P_FDR_* = 0.011) (**Figure** **3**A) and left BA4 (*T* = −2.76, *P_FDR_* = 0.014) (Figure 3B) with right MT+ was significantly reduced in the MDD group. There was no significant group difference in FC between motor network and right MT+ (*T* = −1.04, *P* = 0.301). Moreover, we observed that the FC of right and left BA4 with right MT+ was almost significantly related to the reduced global synchronization, e.g., DC, of both BA4 and MT+ in MDD (see above) and HC (see below) groups (Figure S3, Supporting Information).

We next investigated whether the globally mediated FC of right and left primary motor cortex with the right MT+ is related to psychomotor retardation. Their correlation was indeed significant (*R* = −0.401, *P* = 0.015) (Figure 3C) such that lower BA4 − MT+ FC relates to higher degrees of psychomotor retardation. Moreover, we also saw analogous negative correlation of the FC between left BA4 and right MT+ with the total score of the HAMD (*R* = −0.332, *P* = 0.042) (Figure 3D). For the motor network, its FC with right MT+ was not related to HAMD total score and psychomotor retardation in all its three measures (*P* > 0.05).

### 2.3 Decreased Global Neural Synchronization in Visual MT+ Relates to Psychomotor Retardation

**Figure 4:**
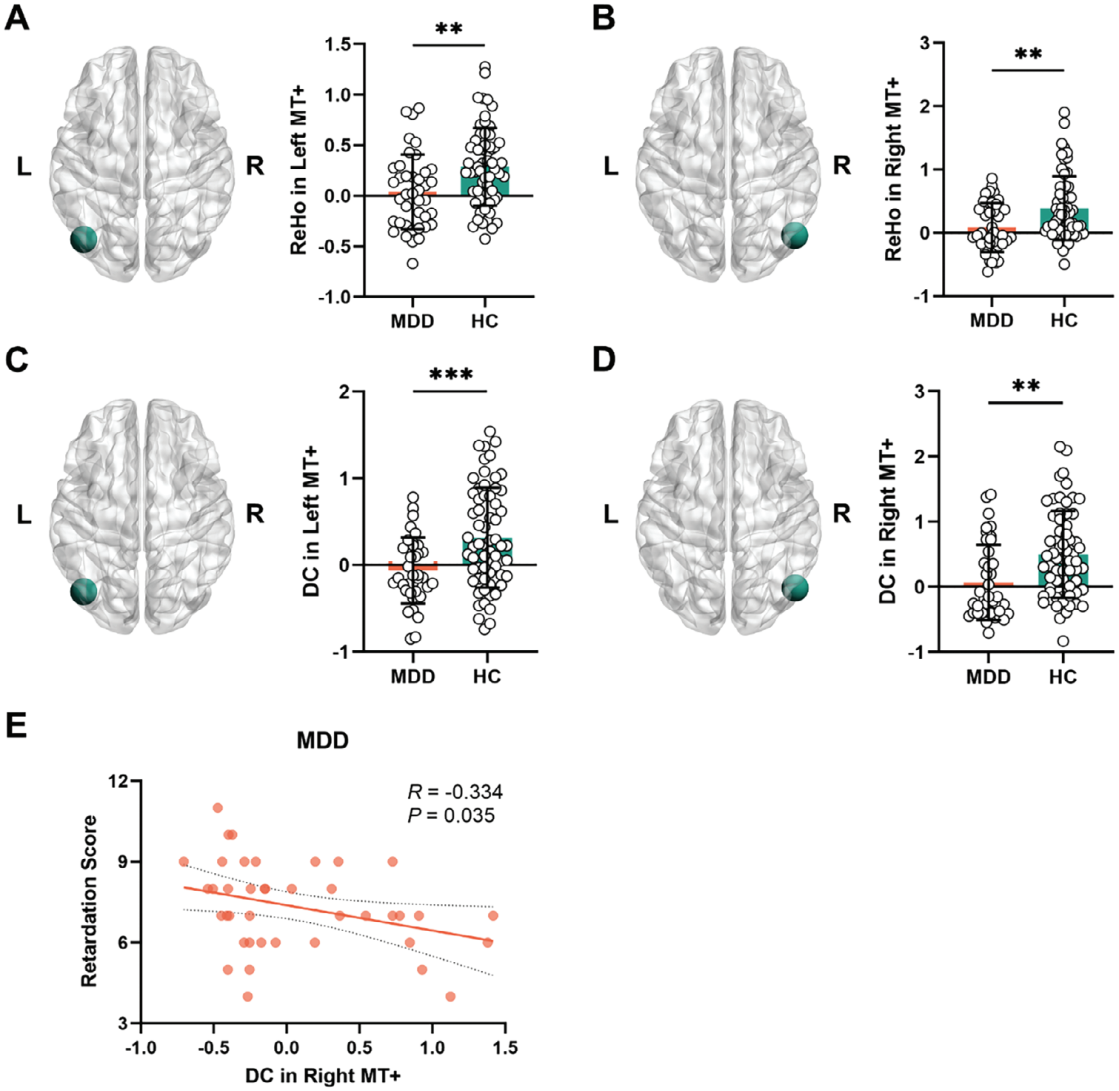
Group differences in DC and ReHo within MT+ between MDD and HC. A–D) MDD shows significantly decreased DC and ReHo in left and right MT+ compared with HC (Student's *t* test). E) Relationship between DC in right MT+ and psychomotor retardation score in MDD group (Pearson correlation). **P_FDR_ < 0.01, ***P_FDR_ < 0.001. L, left; R, right; MT+, middle temporal visual cortex complex; ReHo, regional homogeneity; DC, degree centrality; MDD, major depressive disorder; HC, healthy control.

Given our FC findings, we next ask whether the visual input region MT+ exhibits by itself reduction in both local and global synchronization in MDD. Local regional synchronization, that is, ReHo was decreased in both left (*T* = −3.25, *P_FDR_* = 0.002) (**Figure** **4**A) and right MT+ (*T* = −3.31, *P_FDR_* = 0.003) in MDD (Figure 4B). Similarly, the influence of global synchronization on MT+, e.g., DC, was also significantly reduced in both left (*T* = −3.58, *P_FDR_* < 0.001) (Figure 4C) and right MT+ (*T* = −3.37, *P_FDR_* = 0.001) (Figure 4D) in MDD compared to HC. Moreover, as in BA4, DC positively related to ReHo in both left (*R* = 0.580, *P* = 0.0002) (Figure S4A, Supporting Information) and right MT+ (*R* = 0.583, *P* < 0.0001) (Figure S4B, Supporting Information) of MDD. Similar results were found in the HC group (Figure S4C,D, Supporting Information). Together, these findings show reductions in both global and local neural synchronization in both left and right MT+ in MDD.

Next, we investigated the relationship of the reduced local and global synchronization in MT+ with psychomotor retardation. There was indeed a significant relationship of the reduced DC in right MT+ with the psychomotor retardation score itself (*R* = −0.334, *P* = 0.035) (Figure 4E) while there was no relation to other symptom dimensions in the HAMD scale (Figure S4E, Supporting Information). In contrast to global synchronization, e.g., DC, we did not obtain significant relationship of MT+ local synchronization, e.g., ReHo, with psychomotor retardation (*P* > 0.05).

Together, these findings show that the globally mediated visual cortex MT+ synchronization is reduced in MDD and, even more important, relates to psychomotor retardation. In contrast, local synchronization in MT+, though reduced, did not relate to psychomotor retardation (*P* > 0.05). Furthermore, it shall be mentioned that we tested for the effect of medication load on our results above. We did not observe significant change as it is now mentioned in the Supporting Information, this makes medication effects rather unlikely (see Supporting Information).

### 2.4 Altered MT+ Based Psychophysical Variability in Visual Perception Relates to Psychomotor Retardation

We next raised the question whether visual cortex MT+, in addition to its neural changes, is also altered on the perceptual level. For that purpose, we, applying a standard motion discrimination task as in previous studies,^[^ ^18^, ^23^, ^32^, ^33^ ^]^ examine whether MT+ based psychophysical dynamics as measured by variability is changed in the perception of the MDD subjects.

**Figure 5:**
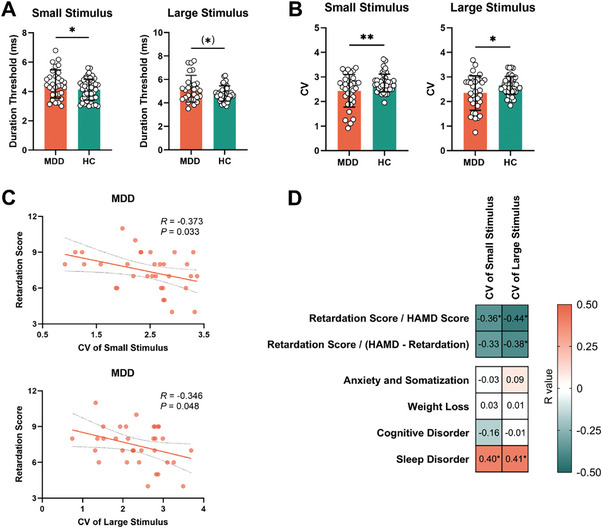
Comparison of the static (duration threshold) and variability (CV) measures of small (diameter of 2°) and large stimuli (diameter of 10°) in MDD and HC groups. A) MDD showed significantly elevated duration threshold of small stimulus compared with HC, but only marginally significant difference in large stimulus (Student's *t* test). B) MDD showed significantly lower CV of small and large stimuli compared with HC (Student's *t* test). C) CV of small and large stimuli related to retardation score (Pearson correlation). D) Relationship between CV of small and large stimuli and all the factors in HAMD‐17 scale (Pearson correlation). (*) *P* < 0.1, *P < 0.05, **P < 0.01. Anxiety/somatization score was the sum of subitems 10, 11, 12, 15, 17 of the HAMD‐17 scale. Weight loss score was the score of subitem 16 of the HAMD‐17 scale. Cognitive disorder score was calculated by summing the scores of subitems 2, 3, 9 of the HAMD‐17 scale. And sleep disorder score was the sum of subitems 4, 5, 6 of the HAMD‐17 scale. MDD, major depressive disorder; HC, healthy control; CV, Coefficient of Variation; HAMD, Hamilton Depression Rating Scale.

We first investigated visual psychophysical performance in HC and MDD in both a standard static and a novel dynamic way. Consistent with our previous study,^[^ ^23^ ^]^ the duration threshold of small stimulus (diameter of 2°) was significantly different between MDD and HC groups (*T* = 2.06, *P* = 0.042) with the MDD subjects showing longer duration. Analogous results, albeit only marginally significant, were obtained in the duration threshold of large stimulus (diameter of 10°) (*T* = 1.96, *P* = 0.054) (**Figure** **5**A). This suggests that the MDD subject need longer time and are thus slower on the perceptual level. Moreover, applying novel dynamic analyses, the MDD group, compared with HC, showed significantly decreased variability, e.g., Coefficient of Variation (CV) of small (*T* = −2.70, *P* = 0.009) and large stimuli (*T* = −2.59, *P* = 0.012) (Figure 5B). This suggests abnormal variability in the MDD subjects’ time series of their psychophysical performance. These results thus show decreased perceptual variability in MDD subjects when processing the visual inputs’ dynamics. Most importantly, the CV of both small (*R* = −0.373, *P* = 0.033) and large stimuli (*R* = −0.346, *P* = 0.048) negatively correlated with psychomotor retardation (Figure 5C). In contrast, the CV did not correlate with other symptom dimensions (anxiety/somatization, weight loss and cognitive disorder) in the HAMD‐17 scale (Figure 5D).

Together, these data show altered MT+ based variability on the visual perceptual level in MDD as manifest in altered temporal features like longer duration and decreased variability. Importantly, these dynamic measures correlated with the severity of psychomotor retardation.

### 2.5 FC between BA4 and MT+ Mediates the Impact of Reduced Global Synchronization in MT+ on Psychomotor Retardation

To further support the key role of reduced globally mediated synchronization of MT+ and BA4 in mediating psychomotor retardation, we probed their trilateral relationship. Firstly, we used Pearson's correlation analysis to investigate how the three variables (DC in right MT+, FC between right BA4 and right MT+, and retardation score) are related to each other in the MDD group. The correlations between DC and FC (*R* = 0.707, *P* < 0.001), DC and retardation score (*R* = −0.382, *P* = 0.028), FC and retardation score (*R* = −0.499, *P* = 0.003) were all significant in MDD patients. This allowed us to apply mediation analyses.

**Figure 6:**
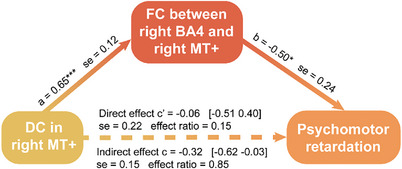
Mediation model analysis in MDD group. FC between right MT+ and right BA4 serves as mediator between DC in right MT+ (predictor) and psychomotor retardation (outcome). *P < 0.05, ***P < 0.001. DC, degree centrality; MT+, middle temporal visual cortex complex; BA, Brodmann Area; FC, functional connectivity.

We then tested the mediation effects in the MDD group (**Figure** **6**). The indirect effect in the mediation model with FC between right MT+ and right BA4 as a mediator is significant whereas the direct effect from DC in right MT+ to retardation score is, by itself, not significant (Direct effect: c’ = −0.06, [−0.51 0.40]; se = 0.22; effect ratio = 0.15; Indirect effect: c = −0.32, [−0.62 −0.03]; se = 0.15; effect ratio = 0.85). This suggests that the globally mediated FC between right MT+ and right BA4 fully mediates the impact of the reduced DC in right MT+ on psychomotor retardation severity. In addition, we also tested using FC as the independent variable and DC as the mediator, this abolished any significant mediation effect (Figure S5, Supporting Information). We did not conduct similar analysis for motor network because there was no significant group difference or correlation with retardation score in FC between motor network and right MT+.

### 2.6 Directed DC and Effective FC in Motor Regions and MT+

**Figure 7:**
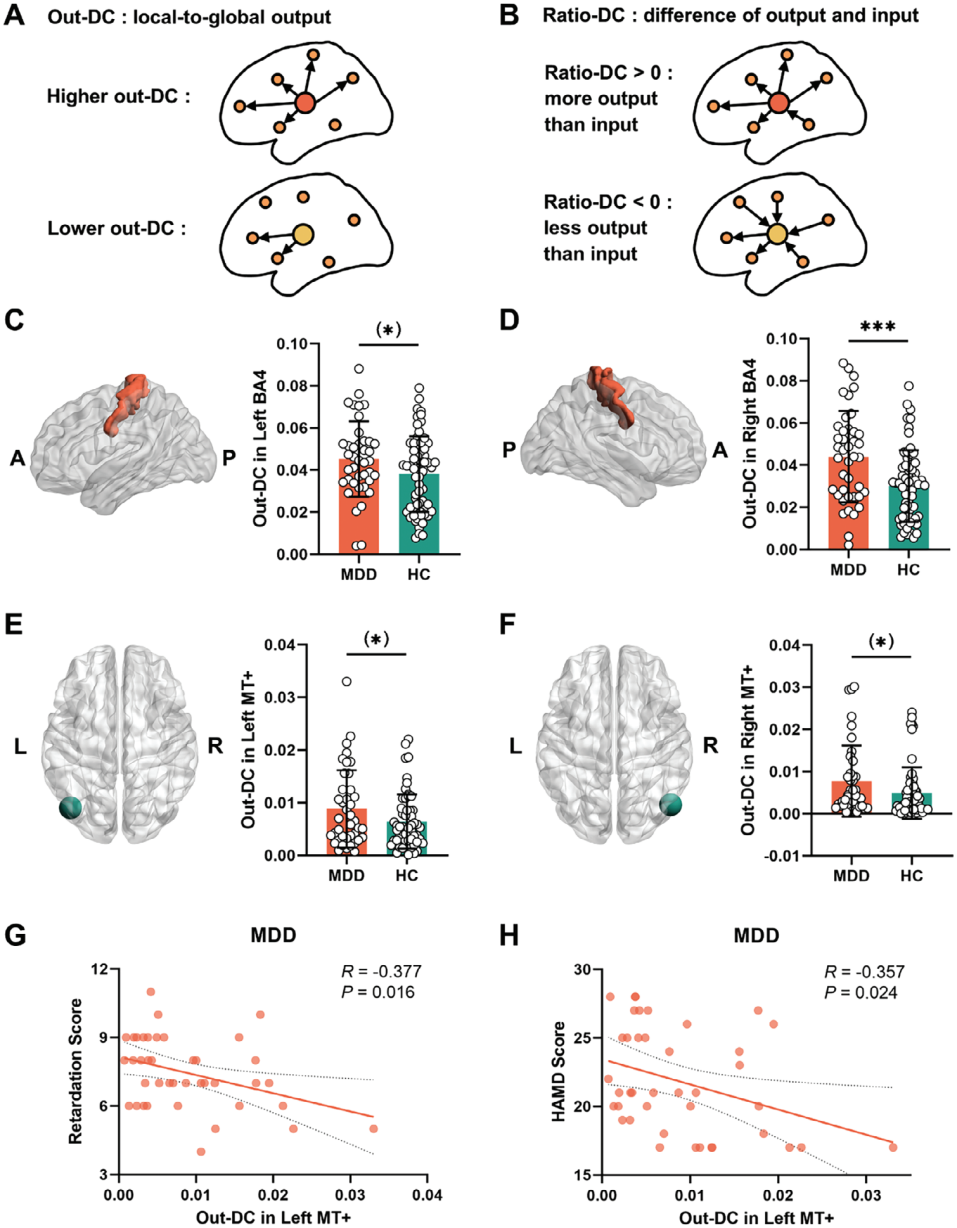
Group differences and relationships of out‐DC in BA4 and MT+. Schematic diagrams of out‐DC A) and ratio‐DC B). C) Out‐DC was nearly significant increased in left BA4 in MDD group (Student's *t* test). D) Increased out‐DC in right BA4 in MDD group (Student's *t* test). Out‐DC was nearly significantly increased in left MT+ E) and right MT+ F) (Student's *t* test). G) Relationship between out‐DC in left MT+ and retardation score in MDD patients (Pearson correlation). H) The correlation between out‐DC in left MT+ and HAMD total score in MDD group (Pearson correlation). Out‐DC, DC in output direction. (*) *P* < 0.01, ***P < 0.001. A, anterior; P, posterior; L, left; R, right; BA, Brodmann Area; MT+, middle temporal visual cortex complex; MDD, major depressive disorder; HC, healthy control.

To analyze the connectivity in a causal way, we conducted Granger causality analysis (GCA) and calculated directed DC (including out‐DC and ratio‐DC, see methods for details) and effective FC in our ROIs. Out‐DC (DC in output direction) represented the effect of the ROI on all other voxels in the brain (**Figure** **7**A). Ratio‐DC (the difference in DC between output and input directions divided by the sum of DC in output and input directions) stood for whether the region operated as source or receiver of information (Figure 7B). Compared to HC group, significant or nearly significant increased out‐DC were found in left BA4 (*T* = 1.98, *P* = 0.050) (Figure 7C), right BA4 (*T* = 3.63, *P* < 0.001) (Figure 7D), and motor network (*T* = 1.90, *P* = 0.061) in MDD group, which were not correlated with HAMD total score or retardation score (*P* > 0.05). Ratio‐DC in left BA4 (*T* = 3.95, *P* < 0.001) (Figure S6B, Supporting Information), right BA4 (*T* = 3.85, *P* < 0.001) (Figure S6C, Supporting Information), and motor network (*T* = 3.05, *P* = 0.003) were increased in MDD patients. Ratio‐DC in left BA4 (*R* = −0.521, *P* < 0.001) (Figure S6D, Supporting Information) and motor network (*R* = −0.405, *P* = 0.009) were significantly related to HAMD total score in MDD group, while ratio‐DC in left BA4, right BA4 and motor network were not correlated with the retardation score (*P* > 0.05).

In left MT+ (*T* = 1.92, *P* = 0.058) (Figure 7E) and right MT+ (*T* = 1.97, *P* = 0.052) (Figure 7F), out‐DC was nearly significantly increased in MDD patients compared to HC group. Relationships between out‐DC in left MT+ and retardation score (*R* = −0.377, *P* = 0.016) (Figure 7G) and HAMD total score (*R* = −0.357, *P* = 0.024) (Figure 7H) were significant in MDD patients. Ratio‐DC was nearly significantly increased in left MT+ (*T* = 1.72, *P* = 0.089) (Figure S6E, Supporting Information), and was increased in right MT+ (*T* = 2.19, *P* = 0.031) (Figure S6F, Supporting Information). Ratio‐DC in left MT+ was related to retardation score (*R* = −0.451, *P* = 0.003) (Figure S6G, Supporting Information). In left MT+ (*R* = −0.409, *P* = 0.008) (Figure S6H, Supporting Information) and right MT+ (*R* = −0.370, *P* = 0.017) (Figure S6I, Supporting Information), ratio‐DC was correlated with HAMD total score.

Finally, unlike undirect FC results, there was no significant group difference in effective FC from MT+ to BA4 or motor network, nor in effective FC from BA4 or motor network to MT+ (*P* > 0.05) (Figure S7, Supporting Information).

### 2.7 Results in the Replication Data Set

All of the above results are from the main data set (i.e., study 1). Similarly, we also computed DC, ReHo, FC, directed DC and effective FC in a replication data set (i.e., study 2). The results in this additional data set were found to be consistent with the existing results in our main data set, further validating the reliability of our findings (Figures S8–S14, Supporting Information).

## 3 Discussion

To the best of our knowledge, this is the first study to examine and directly compare the two main different hypotheses about the source of psychomotor retardation in MDD, motor versus psychomotor, using different clinical‐behavioral assessments and 7 T magnetic resonance imaging (MRI).

Our main findings are: 1) Decreased local and global synchronization and increased local‐to‐global output to the whole brain in primary motor cortex and motor network of MDD without relationship to psychomotor retardation, though. 2) Decreased globally mediated synchronization, e.g., functional connectivity between primary motor cortex and visual cortex MT+ which significantly correlates with the severity of psychomotor retardation. 3) Decreased global synchronization and increased local‐to‐global output to the whole brain in visual cortex MT+ and abnormal MT+ based perceptual variability which both relate to the severity of psychomotor retardation. 4) Decreased global synchronization from visual cortex MT+ through globally mediated FC to primary motor cortex relates to psychomotor retardation. Together these findings support the assumption of a more globally mediated visual to primary motor cortex neural source rather than local regional motor cortex source of psychomotor retardation in MDD. This is well in line with the psychomotor rather than the motor model.

Our first main finding consists in reduced local and global synchronization of the primary motor cortex and motor network in MDD. This is well in line with the various observations of motor cortex activity reduction in MDD.^[^ ^5^, ^10^, ^11^, ^12^ ^]^ Our findings confirm that and extend these results by showing that reduced activity in primary motor cortex and motor network is not only of local regional neural source, that is within the motor region itself as measured by ReHo. Instead, our findings show that the primary motor cortex and motor network are also represented to a lower degree in the cortex’ global activity, e.g., DC. This is in line with various observations of global signal changes and their abnormal regional representation in MDD.^[^ ^16^, ^24^ ^]^ Moreover, MDD showed increased local‐to‐global output to the whole brain from primary motor cortex and motor network, indicating that the regions operate more as source than receiver of information in the brain. However, neither local nor global synchronization measures in primary motor cortex and motor network correlated with the severity of psychomotor retardation. That argues against the motor model in which case one would have expected direct correlation of psychomotor retardation with especially local primary motor cortex and motor network synchronization, e.g., ReHo.

Our next key finding consists in the decrease of globally mediated functional connectivity of primary motor cortex with visual cortex MT+ including its relationship with the severity of psychomotor retardation. While the finding of decreased FC is by itself still compatible with the motor model, the correlation with psychomotor retardation suggests its cortical source beyond and outside the motor cortex itself. Specifically, the degree to which the motor cortex is desynchronized from the global cortex including sensory regions like visual cortex MT+, seems to be key for yielding its relationship to psychomotor retardation. That suggests a neural source of psychomotor retardation outside and beyond the motor cortex in the rest of the global cortex including visual MT+. Moreover, we observed no group difference or correlation with retardation score in effective FC between BA4 and MT+ themselves. This suggests that there may not be a direct connectivity change between MT+ and motor regions in MDD patients, but rather an indirect connectivity change through the involvement of global cortical functional connectivity which, is key for psychomotor retardation.

We therefore next examined where and how the reduced behavioral activity, reflecting decreased variability in psychomotor retardation, is related to outside and beyond the motor cortex itself. Given that various studies show altered visual cortex function in MDD,^[^ ^18^, ^19^, ^20^, ^23^, ^34^ ^]^ we also applied the global synchronization to visual cortex MT+ itself. This, as in the case of the primary motor cortex, shows decreased synchronization of the visual cortex MT+ with the rest of the global cortex in MDD which, on the psychophysical level, is further supported by our finding of decreased perceptual variability in the motion discrimination task.^[^ ^33^ ^]^ Moreover, left MT+ showed increased local‐to‐global output to the whole brain in MDD, and the region operated as source rather than as receiver of information in the brain. That, as in the case of the motor cortex, again suggests a key role of visual cortical activity for global cortical changes.

Most importantly, our three findings of altered visual cortical function, that is, reduced global synchronization of MT+, increased local‐to‐global output from MT+, and MT+ based decreased perceptual variability correlated significantly with the severity of psychomotor retardation.

Given these findings, we suppose a key role of visual input region MT+ on both neural and perceptual levels in mediating the impact of its globally reduced synchronization on both BA4 − MT+ FC and psychomotor retardation. This is indeed further supported by our mediation model showing that the impact of reduced global synchronization of MT+ on psychomotor retardation is mediated by BA4 − MT+ FC. Taken together, we, albeit tentatively, assume that reduced visual input processing in MT+ including its decreased perceptual dynamics, e.g., variability, are key in mediating reduced variability in behavior, e.g., psychomotor retardation, through the decrease in the globally mediated synchronization of MT+ with the primary motor cortex.

As this “locates” the neural source of psychomotor retardation outside the motor regions, our results favor the psychomotor over the motor model. Moreover, it adds a novel psychomotor mechanism to the current list, namely, decreased globally mediated synchronization of visual input and motor output regions.^[^ ^4^ ^]^ We tentatively assume that the decreased synchronization and dynamics in visual input processing on both levels, neural and perceptual, transforms, through its connections with the primary motor cortex, into more or less corresponding decreases in synchronization and dynamics, e.g., variability on the behavioral level, e.g., psychomotor retardation. Reduced synchronization and dynamics or variability may thus be shared by both neural and behavioral levels as their “common currency”.^[^ ^35^, ^36^ ^]^

Some limitations need to be mentioned. While we calculated the retardation scores in different ways with different item numbers on the basis of the HAMD scale (see the Experimental Section), other scales dedicated specifically to psychomotor retardation like the Motor Agitation and Retardation Scale (MARS), the CORE index of melancholia, and the Salpetriere Retardation Rating Scale (SRRS)^[^ ^37^, ^38^, ^39^ ^]^ may want to be used in the future. Medication is also a confounding variable. However, no correlation of the medication load with either of our psychopathological, psychophysical, and neuronal measures was obtained. While this does not completely exclude the impact of medication, the observations make it rather unlikely. Finally, we did not really address the more causal mechanisms of psychomotor retardation beyond associating it with regions outside the motor regions. Hence, more causal measures like dynamic causal modelling and ideally also causal interventions with for instance stimulation in motor or visual cortex^[^ ^8^, ^40^ ^]^ are required to better map the causal mechanisms of the seemingly psychomotor rather than motor model of psychomotor retardation.

Together, we investigated two alternative hypotheses of the neural source of psychomotor retardation in acute MDD, motor versus psychomotor. Our findings show reductions in local regional and global cortical synchronization and local‐to‐global output to the whole brain within the primary motor cortex and motor network themselves which, however, do not relate to psychomotor retardation. Instead, the severity of psychomotor retardation relates to different measures indexing reduced global synchronization and increased local‐to‐global output to the whole brain in visual cortex MT+, reduced MT+ based perceptual dynamics as measured by psychophysical variability, and the globally mediated functional connectivity of MT+ with the primary motor cortex. This favors the psychomotor over the motor model as the neural source of psychomotor retardation. Future more causal investigation of such psychomotor global cortical source including its relationship to visual input processing are warranted. Finally, this opens novel clinical opportunities by using the here demonstrated neuronal measures of the more global neural cortical source of psychomotor retardation for both differential diagnosis and stimulation therapy in MDD.

## 4 Experimental Section

### Participants

This work performed two studies. In the study 1, we recruited 41 MDD patients and 64 age‐ and gender‐matched adult healthy subjects (**Table** **1**). The MDD subjects were recruited from the Hangzhou Seventh People's Hospital. The HC subjects were recruited from Zhejiang University and the surrounding communities through advertisements. They were selected to match the MDD group for gender, age, and sociocultural background. For each participant, the experiment date was within two weeks after recruitment, with the MRI scans and visual psychophysical experiment conducted on the same day. Similarly, we also collected a replication data set (i.e., study 2) of 18 MDD patients and 19 HC who participated in MRI experiments (**Table** **2**). The recruitment of subjects in replication data set was detailed in the Supporting Information.

**Table 1:** Demographic and clinical data of patients in main data set (study 1).

Variables	Healthy controls (*n* = 64)	MDD patients (*n* = 41)	*P* value
Gender (M/F)	32/32	17/24	0.392^a)^
Age, years (SD)	24.1 (2.7)	24.9 (6.1)	0.373^b)^
HAMD‐17 scores (SD)	–	21.7 (3.8)	–
Retardation scores (SD)	–	7.4 (1.6)	–
Treatment, *n* (%)			
Antidepressants		30 (73.2)	
Antipsychotics		18 (43.9)	
Mood stabilizers		10 (24.4)	
Benzodiazepines		26 (63.4)	

Abbreviations: HAMD, Hamilton Depression Rating Scale; MDD, major depressive disorder; SD, standard deviation.

^a)^ Chi‐square test;

^b)^ Student's *t*‐test. Retardation score was calculated by summing the scores of subitems 1, 7, 8, 14 of the HAMD‐17 scale.

**Table 2:** Demographics and patient clinical data in replication data set (study 2).

Variables	Healthy controls (*n* = 19)	MDD patients (*n* = 18)	*P* value
Gender (M/F)	8/11	3/15	0.091^a)^
Age, years (SD)	23.3 (2.7)	25.2 (6.2)	0.240^b)^
HAMD‐17 scores (SD)	–	25.2 (2.5)	–
Retardation scores (SD)	–	9.6 (1.4)	–

Among the 18 MDD patients, only 14 have HAMD‐17 scale data.

Abbreviations: HAMD, Hamilton Depression Rating Scale; MDD, major depressive disorder; SD, standard deviation.

^a)^ Chi‐square test;

^b)^ Student's *t*‐test.

All subjects had an education background above the college degree, and had normal or corrected to normal vision. Inclusion criteria of the MDD subjects were: i) presence of an acute depressive episode and the diagnosis MDD in accordance with the Diagnostic and Statistical Manual of Mental Disorders, Fifth Edition (DSM‐V) as a) established by the assessing psychiatrist, and b) confirmed with Mini International Neuropsychiatric Interview (M.I.N.I.);^[^ ^41^ ^]^ ii) clinical symptoms of depression as measured by a Hamilton Depression Rating Scale (HAMD‐17) ≥ 17; and iii) MDD subjects treated with the agent of selective serotonin reuptake inhibitors (SSRI) if they are taking medications. Exclusion criteria of MDD subjects were: i) any other psychiatric disorder, or a mental disorder caused by a physical illness or substance abuse or a personality disorder; ii) history of traumatic brain injury, epilepsy, or other known organic lesion of the central nervous system; iii) presence of psychotic symptoms during the depressive episodes; iv) history of endocrine disease or blood, heart, liver, kidney dysfunction, another medical disorder such as diabetes, or pregnancy; and v) individuals with claustrophobia or those with metal implants in their bodies, which make them unsuitable for MRI experiments. Inclusion criterion of the HC subjects was: i) no personal or two‐lineage, three‐generation family history of psychiatric or mental disorder. The exclusion criteria of HC participants were the same as items ii, iii, iv, and v of the exclusion criteria for MDD subjects. The study 1 was approved by the Ethics committee of Hangzhou Seventh People's Hospital. All participants gave written informed consents.

The majority of the acute MDD patients in our main sample were taking medications, including mood stabilizers, antipsychotics, antidepressants, and benzodiazepines, which may possibly influence results. Following recent suggestions and standards,^[^ ^42^ ^]^ we examined the potential impact of the psychotropic medication load—the number and dosage of different medications, we then used the codes 0, 1, 2, and 3 to indicate no medication, and dose‐equivalents below, equal, or above the mean effective daily dose, respectively.^[^ ^43^ ^]^ A composite measure of the medication load was generated by summing all individual medication codes for each category and each individual MDD subject. We explored how medications might affect resting state fMRI (rsfMRI) and behavioral data. We achieved this by correlating the pharmacological load of the medications with retardation score and various metrics from imaging, psychophysical paradigm. Additionally, an independent sample *t*‐test was employed to compare these variables across different classes of medication (details in Supporting Information).

### Factors in HAMD‐17 Scale

Factors of HAMD‐17 scale reflected different characteristics in depression. There were five main factors: psychomotor retardation, anxiety/somatization, weight loss, cognitive disorder and sleep disorder. Psychomotor retardation score was calculated by summing the scores of subitems 1, 7, 8, 14 of the HAMD‐17 scale. Anxiety/somatization score was the sum of subitems 10, 11, 12, 15, 17 of the HAMD‐17 scale. Weight loss score was the score of subitem 16 of the HAMD‐17 scale. Cognitive disorder score was calculated by summing the scores of subitems 2, 3, 9 of the HAMD‐17 scale. And sleep disorder score was the sum of subitems 4, 5, 6 of the HAMD‐17 scale.

### Measurement of Visual Motion Perception

All stimuli were generated using Psychophysics Toolbox^[^ ^44^ ^]^ based on MATLAB (MathWorks, Natick, MA, USA) and were shown on a linearized monitor (1920 × 1080 resolution, 100‐Hz refresh rate, Cambridge Research System, UK). The viewing distance was 72 cm from the screen, with the head stabilized by a chinrest. Stimuli were drawn against a gray (56 cd m^−2^) background.

The details of the procedure for measurement are available in our recent studies.^[^ ^18^, ^23^ ^]^ Briefly, large (diameter of 10°) and small (diameter of 2°) stimuli were vertically drifting sinusoidal gratings with high contrast (contrast: 50%; spatial frequency, 1 cycle/°; speed, 4°/s) (see Figure S1 in Song et al. (2021)).^[^ ^23^ ^]^ The edge of the grating was blurred with a raised cosine function (width, 0.3°). The grating was ramped on and off with a Gaussian temporal envelope, and the grating duration was defined as 1 standard deviation (SD) of the Gaussian function. The duration was adaptively adjusted in each trial, and duration thresholds were estimated by a staircase procedure. Thresholds for large and small gratings were obtained from a 160‐trial block that contained four interleaved three‐down/one‐up staircases. Therefore, the movement of the grating is constantly changing from trial to trial based on the subject's judgment, resulting in dynamic visual input. Stimulus demonstration and practice trials were presented before the first run. Auditory feedback was provided for each wrong response.

### Static and Dynamic Analysis of Psychophysical Performance

The psychophysical experiment recorded the grating duration of each trial, forming a time series. For each participant, we computed the correct rate for different stimulus durations separately for each stimulus size. These values were then fitted to a cumulative Gaussian function, and the static duration threshold corresponding to the 75% correct point on the psychometric function was estimated for each stimulus size.

In order to reflect perceptual dynamics of visual input, CV was computed for small and large stimuli of each subject based on the duration time series. To capture the variability between trials, a new time series was derived by calculating the absolute value of the difference between the grating durations of all neighboring trial, excluding the consecutively presented maximum and minimum values at the experiment's outset. Then, the mean value and standard deviation which reflect the variability in trial‐by‐trial grating durations were calculated based on the new time series. Finally, CV was defined by standard deviation divided by mean value. It reflects the variability of the trials’ duration relative to the overall mean across all trials

(1) $$Mean\;=\frac{1}{N-1}\;\sum_{i\;=\;2}^{N}\left|X\left[i\right]-X\left[i-1\right]\right|$$

(2) $$SD\;=\sqrt{\;\frac{1}{N-1}\sum_{i=2}^{N}{\left(\left|X\left[i\right]-X\left[i-1\right]\right|-Mean\right)}^{2}}$$

(3) $$\mathrm{CV}\;=\frac{SD}{Mean}$$

where *N* is the length of time series *X*. In this study, the grating duration of large or small stimuli constitute the time series *X*, with *N* value of 80. Continuous ceiling values are considered irrational, and these difference elements are removed.

### MRI Data Acquisition

MRI experiments were performed in a 7 T whole body MR system (Siemens Healthcare, Erlangen, Germany) with a Nova Medical 32 channel array head coil. Session included rsfMRI and structural MRI (sMRI). rsfMRI scans were acquired with 1.5 mm isotropic resolution (transverse orientation, TR/TE = 2000/20.6 ms, 160 volumes, slice number = 110, flip angle = 70°, multi‐band acceleration factor = 5, eyes closed, 6 minutes and 24 seconds). sMRI scans were obtained using a MP2RAGE sequence (TR/TI1/TI2 = 5000/901/3200 ms, 8 min and 42 s) with 0.7‐mm isotropic resolution. MRI data acquisition for the replication data set are described in the Supporting Information.

### rsfMRI Data Processing and Analysis

rsfMRI data preprocessing processes for main sample and replication sample were the same, performed in Statistical Parametric Mapping 12 (SPM 12, http://www.fil.ion.ucl.ac.uk/spm/) using the Data Processing and Analysis for Brain Imaging (DPABI) toolbox^[^ ^45^ ^]^ including: removal of the volumes in first 10 seconds, realignment, coregistration of anatomical and functional images for each subject, segmentation of the anatomical images using Diffeomorphic Anatomical Registration through Exponentiated Lie algebra algorithm (DARTEL), linear detrend, nuisance covariates regression (with realignment Friston 24‐parameter, white matter and CSF signal),^[^ ^46^ ^]^ normalization to the standard Montreal Neurological Institute (MNI) space with a resolution of 1.5 mm^3^ using DARTEL, spatial smoothing with a 3‐mm full width at full‐wide‐half‐maximum (FWHM) Gaussian kernel, and band‐pass filtering with standard frequency band (SFB, 0.01–0.1 Hz). Subjects with head movements greater than 3 mm or 3° were excluded. Since smoothing can blur the relationships between adjacent voxels and reduce the precision of voxel signals, which will negatively affect the results of ReHo, undirected DC and directed DC, smoothing is not performed in the preprocessing before calculating ReHo, undirected DC and directed DC.

Before time series extraction, we defined the regions of interests (ROIs). Left and right primary motor cortex were defined by Brodmann Area 4 (BA4) mask, which was obtained from the Brodmann template. Motor network was defined as the union of brain regions associated with motor function (Figure S15, Supporting Information),^[^ ^5^ ^]^ including bilateral primary motor cortex (Brodmann Area 4), primary sensory cortex (Brodmann Area 1, 2, and 3), superior parietal lobule, thalamus, caudate, putamen, pallidum, and supplementary motor area (SMA). Primary motor and sensory cortices were from Brodmann atlas, and the remaining regions were from the Harvard‐Oxford atlases.^[^ ^47^, ^48^, ^49^, ^50^ ^]^ Left MT+ and right MT+ were each defined by a cytoarchitectonically probabilistic map.^[^ ^51^ ^]^ After preprocessing, we took the left BA4, right BA4, motor network as well as the left and right MT+ as ROIs and correlated their time series with each other (i.e., functional connectivity, FC). All the FC values were Fisher‐Z‐transformed.

Local regional neuronal synchronization was calculated by ReHo for each subject using DPABI. Specifically, for each voxel, Kendall's coefficient of concordance was calculated between the time series for the specified voxel and those of its 26 nearest neighbors,^[^ ^52^, ^53^ ^]^ generating a voxel‐wised ReHo map for each subject. The individual ReHo map was standardized into subject‐level z‐score maps by subtracting the mean voxel‐wise ReHo obtained for the entire brain (global mean of ReHo) then dividing by the standard deviation across voxels.^[^ ^53^, ^54^ ^]^ After this, a spatial smoothing of 3‐mm was performed on the ReHo map.^[^ ^54^ ^]^ We extracted the ReHo values within left BA4, right BA4, motor network as well as for left and right MT+ for each subject.

Global neuronal synchronization was calculated using DC^[^ ^24^ ^]^ whose analysis was performed for each subject using DPABI. Specifically, voxel‐based graphs were generated for each subject, where the correlation between the time series of each voxel with every other voxel of the brain was calculated. A weighted, undirected adjacency matrix was then obtained by thresholding each correlation at *R* > 0.25. Based on the graph, DC was calculated at the individual level.^[^ ^55^ ^]^ DC was computed as the number of meaningful functional connections (positive correlations) between each voxel within the region of interest and all other voxels in the rest of the brain. In addition, normalized DC indices were calculated by transforming DC to *z*‐scores based on the global mean of DC and standard deviation across voxels for the entire brain.^[^ ^55^, ^56^ ^]^ After this, a spatial smoothing of 3 mm was performed on the DC map.^[^ ^55^, ^57^ ^]^ The DC value within left BA4, right BA4, motor network as well as left and right MT+ were extracted for each subject.

### Effective FC and Directed DC Calculation

GCA was used to describe the causal relationship between time series of ROIs based on the Resting‐State fMRI Data Analysis Toolkit plus (RESTplus V1.24, http://restfmri.net/forum/ restplus).^[^ ^58^ ^]^ As a definition from Granger in the field of economics,^[^ ^59^ ^]^ if the time series of x could predict the time series of y, y is said to have a causal influence on x. X and y can be a region or voxel in the brain. Signed‐path coefficient of bivariate ROI‐wise GCA was used to reflect the effective FC between ROIs.

Bivariate voxel‐wise GCA with BA4, motor network and MT+ as the ROIs were also performed and GCA signed‐path coefficient maps were generated. These maps reflected the output functional connections from each ROI to all other voxels in the rest of the brain and input functional connections to each ROI. Similar to the calculation of undirected DC, out‐DC was computed as the number of meaningful functional connections (signed‐path coefficient > 0.25) from each ROI to all other voxels in the rest of the brain. In‐DC was calculated as the number of meaningful functional connections (signed‐path coefficient > 0.25) from all other voxels in the rest of the brain to each ROI. Simply put, out‐DC and in‐DC respectively represented the global output and input of the ROI. Ratio‐DC was defined as the difference between out‐DC and in‐DC divided by the sum of out‐DC and in‐DC. It stood for whether the region operated as source or receiver of information

(4) $$ratio\_DC\;=\frac{out\_DC-in\_DC}{out\_DC+in\_DC}$$

In Equation (4), out‐DC, in‐DC and ratio‐DC were written as “out_DC,” “in_DC,” and “ratio_DC” to avoid confusion between hyphens and minus signs. Out‐DC and ratio‐DC were included in the subsequent statistical analysis because we wanted to study the ability of ROIs to act as a neural source.

### Statistical Analysis

In the study, outliers were defined as values that exceed 1.5 times the interquartile range and removed before statistical analysis. SPSS 26 (IBM, USA) was used to conduct statistical analysis in the study. Two‐tailed Student's t test was used to examine the differences between MDD group and healthy control (HC). Two‐tailed Pearson's correlation coefficients were calculated to analyze the relationship between indicators. Differences or correlations were considered statistically significant if *P* < 0.05. In cases where multiple comparisons were made, false discovery rate correction was used to adjust *P* value. PROCESS version 3.4, a toolbox in SPSS 26, was used to examine the mediation model. There are some prerequisites for mediation analysis: the independent variable should be a significant predictor of the mediator, and the mediator should be a significant predictor of the dependent variable. We examined whether the association between DC in right MT+ and retardation score was mediated by FC between right BA4 and right MT+. Bias‐corrected bootstrap methodology was used to evaluate the significance of the direct and indirect effect, in which 95% confidence interval (CI) was calculated with 5000 independent samples. If the bootstrap 95% CI of indirect effect did not contain zero, it indicated a significant mediating effect in the model.

## Conflict of Interest

The authors declare no conflict of interest.

## Author Contributions

X.M.S. and D.L. contributed equally to this work. X.M.S., D.L., and G.N. designed the framework and the logic of the analysis. X.M.S. and D.L. conducted human experiments, analyzed data, and D.L. created figures. X.H., J.H., and Z.T. were in charge of the patients’ recruitment and assessment. D.Y. guided partial data analysis. G.N. together with X.M.S., D.L. and D.H. wrote the manuscript. A.W.R. edited the manuscript. All authors evaluated and approved the final version of the manuscript.

## Supporting information

Supporting Information

## Data Availability

The data that support the findings of this study are available from the corresponding author upon reasonable request.
