# Supplementary material for: Motor versus Psychomotor? Deciphering the Neural Source of Psychomotor Retardation in Depression
Source: Adv Sci (Weinh). 2024 Aug 29;11(40):2403063. doi: 10.1002/advs.202403063 (PMC11515905; doi:10.1002/advs.202403063)
Supplement: Supplementary file 1 — Supporting Information [file ADVS-11-2403063-s001.docx]

Supporting Information

Motor vs Psychomotor? Deciphering the Neural Source of Psychomotor Retardation in Depression

Xue Mei Song*, Dong-Yu Liu, Dusan Hirjak, Xi-Wen Hu, Jin-Fang Han, Anna Wang Roe, De-Zhong Yao, Zhong-Lin Tan*, Georg Northoff*

**Supporting Methods**

**Participants in Replication Data Set**

In the study 2, the MDD subjects were also recruited from the Hangzhou Seventh People’s Hospital. The HC subjects were recruited from Hangzhou Normal University and the surrounding communities through advertisements. For each participant, the MRI experiment date was within two weeks after recruitment. All subjects had an education background above the college degree. Inclusion criteria of the MDD subjects were: (i) presence of an acute depressive episode and the diagnosis MDD in accordance with the Diagnostic and Statistical Manual of Mental Disorders, Fifth Edition (DSM-V) as (a) established by the assessing psychiatrist, and (b) confirmed with Mini International Neuropsychiatric Interview (M.I.N.I.). The exclusion criteria of MDD subjects and the recruitment of HC subjects were the same with main data set (i.e., study 1). The study 2 was approved by the Ethics committee of Hangzhou Seventh People’s Hospital. All participants gave written informed consents.

**MRI Data Acquisition for Replication Data Set**

MRI experiments were performed in a 7 T whole body MR system (Siemens Healthcare, Erlangen, Germany) with a Nova Medical 32 channel array head coil. Session included resting-state fMRI (rsfMRI) and structural MRI (sMRI). rsfMRI scans were acquired with 1.5-mm isotropic resolution (transverse orientation, TR/TE = 1000/22.8 ms, 360 volumes, slice number = 95, flip angle = 45°, multi-band acceleration factor = 5, eyes closed, 6 minutes and 34 seconds). sMRI scans were obtained using a MPRAGE sequence (TR/TI1 = 2590/1050 ms, 6 minutes and 14 seconds) with 0.75-mm isotropic resolution.

**Testing the Effect of Medication Load on Variables in rsfMRI and Psychophysical Data and Pathological Symptom**

We investigated the potential impact of medications on resting state fMRI data by correlating the resulting pharmacological load with ReHo and DC in left BA4, right BA4 and motor network, FC between BA4 and right MT+, ReHo and DC in right MT+, out-DC and ratio-DC in BA4, motor network and MT+, CV of small and large stimuli, and retardation score. Then, to further control for an eventual effect of pharmacotherapy on the ReHo and DC in left BA4, right BA4 and motor network, FC between BA4 and right MT+, ReHo and DC in right MT+, out-DC and ratio-DC in BA4, motor network and MT+, CV of small and large stimuli, and retardation score, we compared these variables by using an independent sample t test for each medication class (mood stabilizers, antidepressants, benzodiazepines, and antipsychotics), between those patients who were in treatment with the respective drug and those who were not.

**Supporting Results**

**Results in the Replication Data Set**

In replication data set (i.e., study 2), compared to HC group, ReHo in left BA4 (*T* = -5.02, *P_FDR_* < 0.0001) (Figure S8A), right BA4 (*T* = -5.28, *P_FDR_* < 0.0001) (Figure S8B) and motor network (*T* = -4.82, *P* < 0.001) (Figure S9A) were significantly decreased in MDD group. And DC also significantly reduced in left BA4 (*T* = -4.48, *P_FDR_* < 0.0001) (Figure S8C), right BA4 (*T* = -4.49, *P_FDR_* < 0.0001) (Figure S8D) and motor network (*T* = -4.44, *P* < 0.001) (Figure S9B). Furthermore, DC positively correlated ReHo in both left BA4 (*R* = 0.869, *P* < 0.0001) (Figure S8E), right BA4 (*R* = 0.740, *P* = 0.001) (Figure S8F) and motor network (*R* = 0.678, *P* = 0.002) (Figure S9C) of MDD in replication data set. Similar results were found in the HC group (*P* < 0.01) (Figure S8G, S8H, S9D) in replication data set.

To investigate the synchronization between BA4 and MT+, we also computed BA4 – MT+ FC in the replication set. Compared to HC group, FC of both right (*T* = -2.84, *P_FDR_* = 0.015) (Figure S10A) and left BA4 (*T* = -2.58, *P_FDR_* = 0.015) (Figure S10B) with right MT+ was significantly reduced in the MDD group. There was no significant group difference in FC between motor network and left MT+ (*T* = -1.54, *P_FDR_* = 0.133) and right MT+ (*T* = -2.27, *P_FDR_* = 0.058).

Compared to HC group, ReHo significantly decreased in left (*T* = -2.74, *P_FDR_* = 0.020) (Figure S11A) and right MT+ (*T* = -2.34, *P_FDR_* = 0.025) (Figure S11B) in MDD group. In left MT+, DC significantly reduced in MDD group (*T* = -3.83, *P_FDR_* = 0.001) (Figure S11C). And ReHo in right MT+ marginally decreased in MDD group (*T* = -1.84, *P_FDR_* = 0.075) (Figure S11D). Similar to BA4 and motor network, DC and ReHo in the left (*R* = 0.713, *P* = 0.002) (Figure S11E) and right MT+ (*R* = 0.580, *P* = 0.012) (Figure S11F) were significantly correlated in the MDD group, with similar results observed in the HC group as well (*P* < 0.01) (Figure S11G, H).

We also conducted Granger causality analysis and calculated directed DC (including out-DC and ratio-DC) and effective FC in replication data set. Compared to HC group, significant increased out-DC were found in left BA4 (*T* = 6.05, *P* < 0.001) (Figure S12A), right BA4 (*T* = 5.85, *P* < 0.001) (Figure S12B), and motor network (*T* = 4.74, *P* < 0.001) in MDD group. Ratio-DC in left BA4 (*T* = 2.10, *P* = 0.044) (Figure S13A) and right BA4 (*T* = 2.61, *P* = 0.013) (Figure S13B) were increased in MDD patients.

In left MT+ (*T* = 4.49, *P* < 0.001) (Figure S12C) and right MT+ (*T* = 4.23, *P* < 0.001) (Figure S12D), out-DC was increased in MDD patients compared to HC group. Increased ratio-DC was found in left MT+ (*T* = 2.36, *P* = 0.024) (Figure S13C) and right MT+ (*T* = 2.61, *P* = 0.013) (Figure S13D).

Different from undirect FC results, in replication data set, there was no significant group difference in effective FC from MT+ to BA4 or motor network, nor in effective FC from BA4 or motor network to MT+ (*P* > 0.05) (Figure S14).

**Medication Load not Significantly Affect rsfMRI and Psychophysical Data and Psychomotor Retardation Score**

In correlation analysis, ReHo and DC in left BA4, right BA4 and motor network, FC between BA4 and right MT+, ReHo and DC in right MT+, out-DC and ratio-DC in BA4, motor network and MT+, CV of small and large stimuli, and retardation score did not relate to the medication load code of antidepressants, antipsychotics, mood stabilizers and benzodiazepines (P_FDR_ > 0.05). Then, in the independent t-test, we found no significant difference between patients who were in treatment with antidepressants (n = 30) and patients who were not (n = 11) (P_FDR_ > 0.05), between patients who were in treatment with antipsychotics (n = 18) and patients who were not (n = 23) (P_FDR_ > 0.05), between patients who were in treatment with mood stabilizers (n = 10) and patients who were not (n = 31) (P_FDR_ > 0.05), as well as between patients who were in treatment with benzodiazepines (n = 26) and patients who were not (n = 15) (P_FDR_ > 0.05).

**Supporting Figures:**


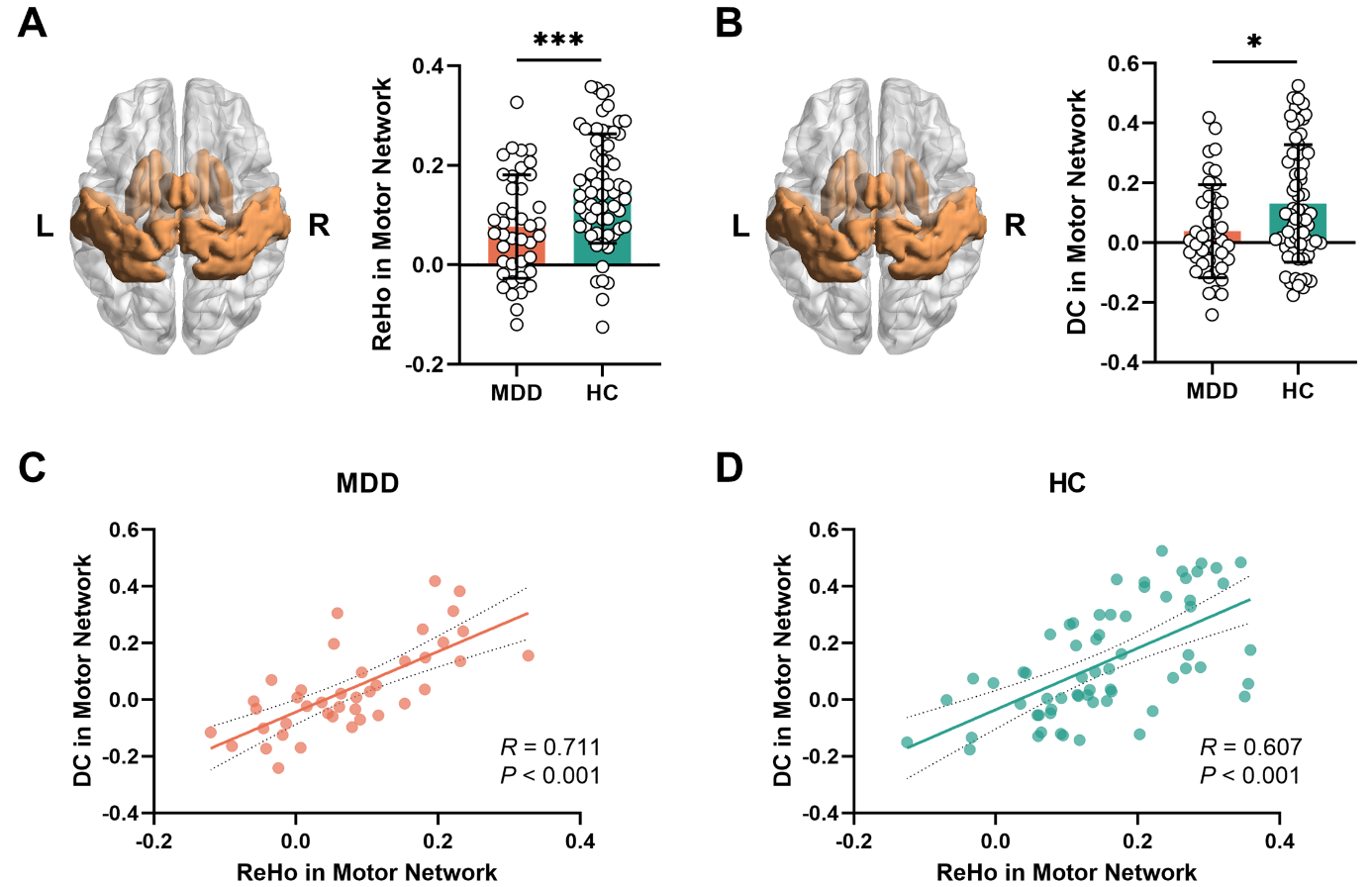


**Figure S1.** Group differences and relationships in ReHo and DC within motor network. Compared to HC group, MDD showed reduced ReHo (A) and DC (B) in motor network (Student’s *t* test). Correlation between ReHo and DC in motor network in MDD (C) and HC (D) groups (Pearson correlation). * *P* < 0.05, *** *P* < 0.001. L, left; R, right; MDD, major depressive disorder; HC, healthy control.


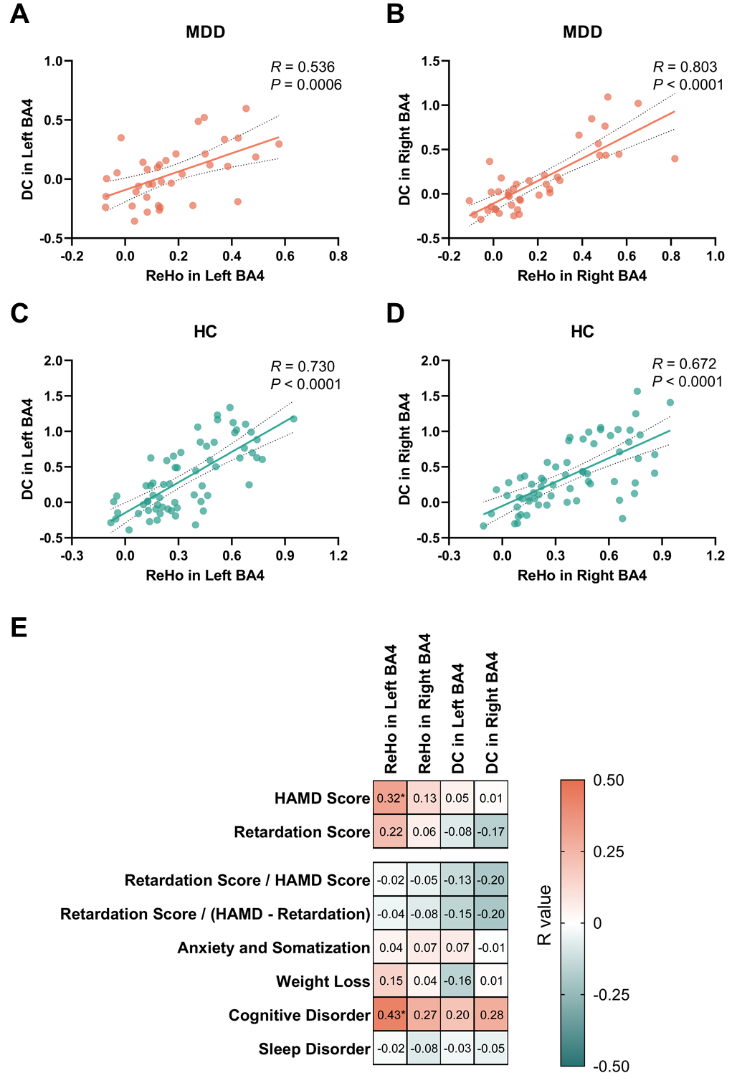


**Figure S2.** Relationship of DC and ReHo within BA4 in MDD and HC. DC correlated with ReHo within BA4 in MDD (A and B) and HC group (C and D) (Pearson correlation). E) Correlation between DC, ReHo within left and right BA4 and symptom severity in MDD group (Pearson correlation). * *P_FDR_* < 0.05.

Anxiety/somatization score was the sum of subitems 10, 11, 12, 15, 17 of the HAMD-17 scale. Weight loss score was the score of subitem 16 of the HAMD-17 scale. Cognitive disorder score was calculated by summing the scores of subitems 2, 3, 9 of the HAMD-17 scale. And sleep disorder score was the sum of subitems 4, 5, 6 of the HAMD-17 scale. DC, degree centrality; ReHo, regional homogeneity; BA, Brodmann Area; MDD, major depressive disorder; HC, healthy control; HAMD, Hamilton Depression Rating Scale.


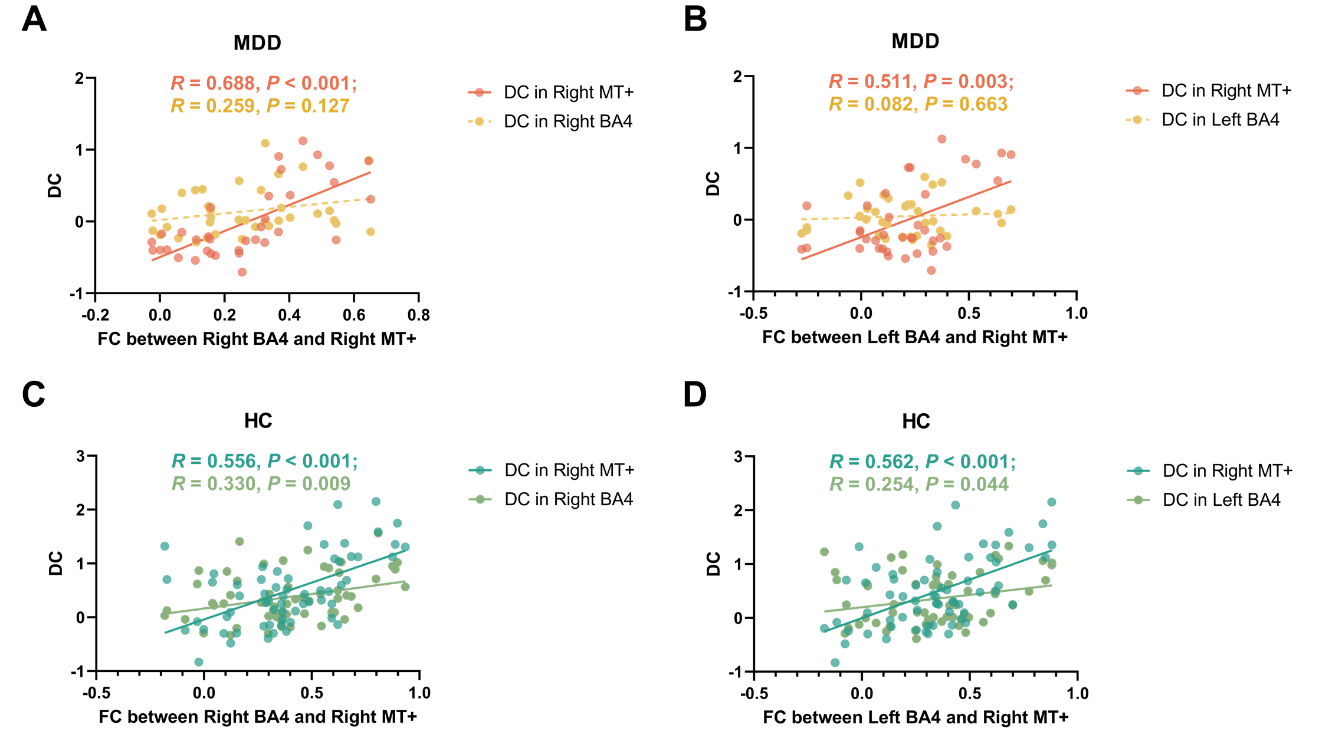


**Figure S3.** Relationship of FC between BA4 and right MT+ and DC in BA4 and right MT+. FC between right (A) and left BA4 (B) and right MT+ were significantly correlated with DC in right MT+ in MDD group, although FC and DC in BA4 were not significantly correlated (Pearson correlation). In HC group, FC between right (C) and left BA4 (D) and right MT+ were significantly related to DC in both BA4 and right MT+ (Pearson correlation). DC, degree centrality; FC, functional connectivity; BA, Brodmann Area; MT+, middle temporal visual cortex complex.


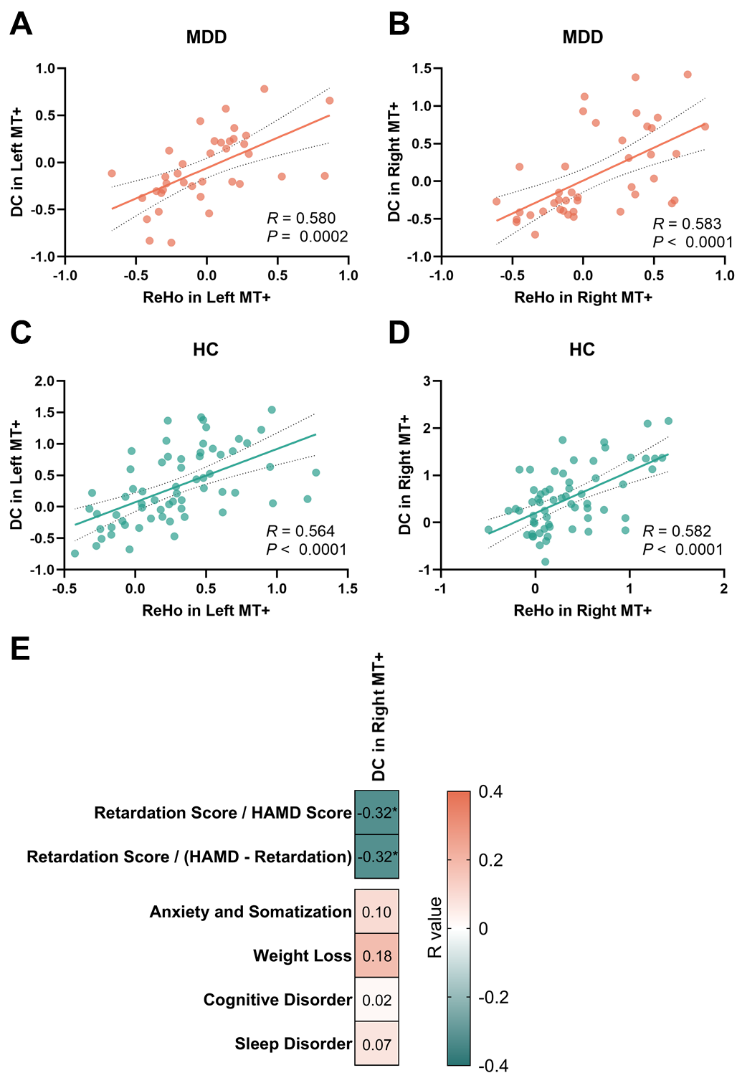


**Figure S4.** Relationship of DC and ReHo within MT+ in MDD and HC. DC significantly related ReHo within MT+ in MDD (A and B) and HC group (C and D) (Pearson correlation). E) Correlation between DC in right MT+ and symptom severity in MDD group (Pearson correlation). * *P_FDR_* < 0.05. DC, degree centrality; ReHo, regional homogeneity; MT+, middle temporal visual cortex complex; MDD, major depressive disorder; HC, healthy control; HAMD, Hamilton Depression Rating Scale.


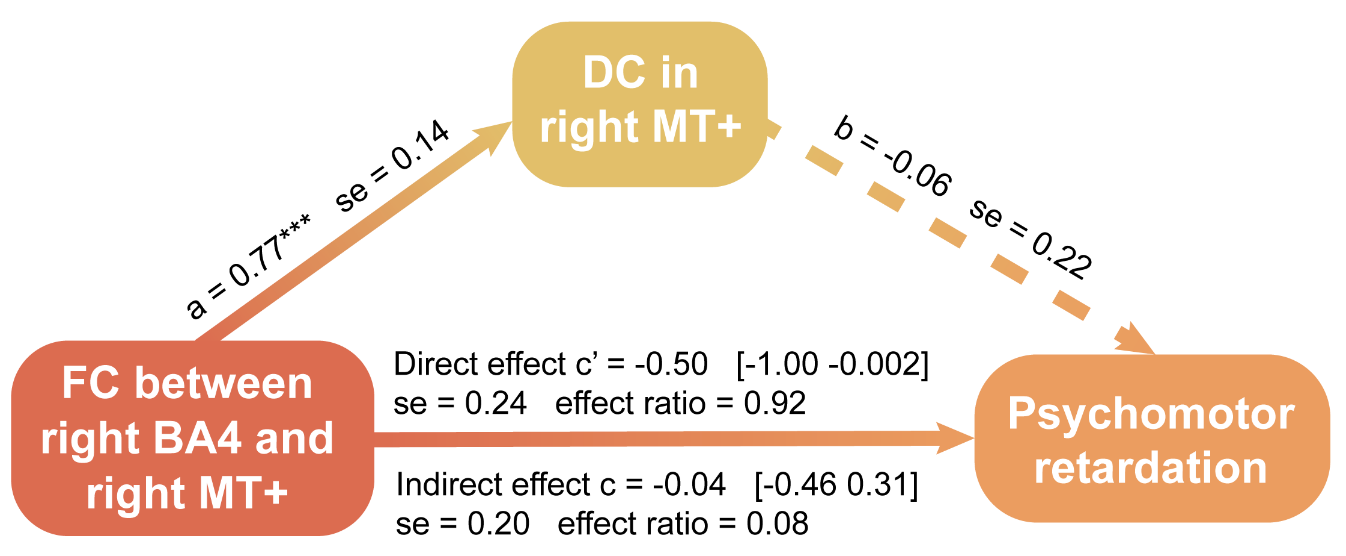


**Figure S5.** Mediation effect analysis in MDD group when DC was used as mediator between FC (predictor) and psychomotor retardation (outcome). *** *P* < 0.001. FC, functional connectivity; BA, Brodmann Area; MT+, middle temporal visual cortex complex; DC, degree centrality.


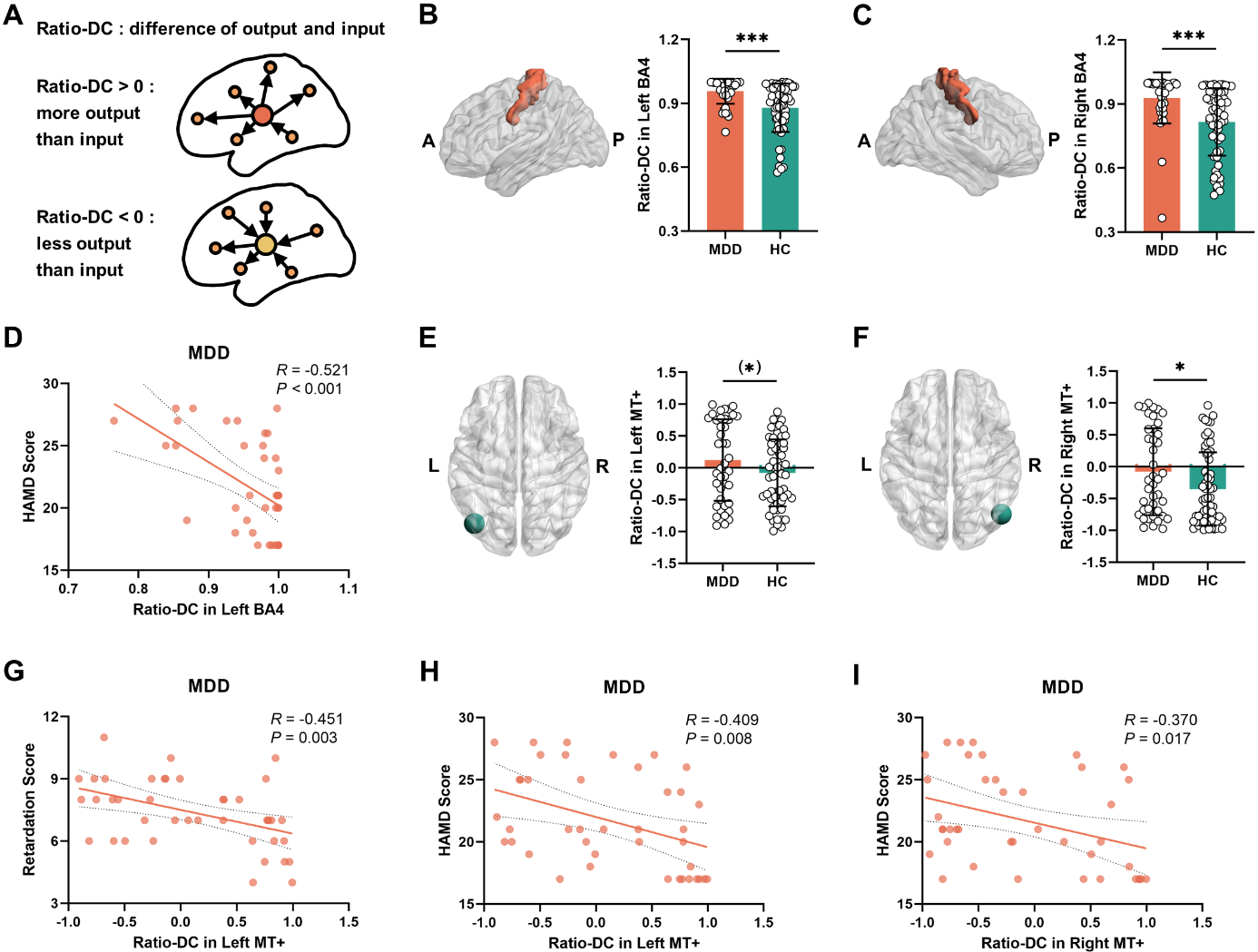


**Figure S6.** Comparison and relationships in ratio-DC within BA4 and MT+. A) Diagram of ratio-DC. B) Ratio-DC was significant increased in left BA4 in MDD group (Student’s *t* test). C) Increased ratio-DC in right BA4 in MDD group (Student’s *t* test). D) Correlation between ratio-DC in left BA4 and HAMD total score in MDD (Pearson correlation). E) Ratio-DC was nearly significantly increased in left MT+ in MDD group (Student’s *t* test). F) Increased ratio-DC in right MT+ in MDD group (Student’s *t* test). G) Correlation between ratio-DC in left MT+ and retardation score in MDD patients (Pearson correlation). Relationships between ratio-DC in left MT+ (H) and right MT+ (I) and HAMD total score in MDD group (Pearson correlation). Ratio-DC, the difference in DC between output and input directions divided by the sum of DC in output and input directions. (*) *P* < 0.01, * *P* < 0.05, *** *P* < 0.001. A, anterior; P, posterior; L, left; R, right; BA, Brodmann Area; MT+, middle temporal visual cortex complex; MDD, major depressive disorder; HC, healthy control.


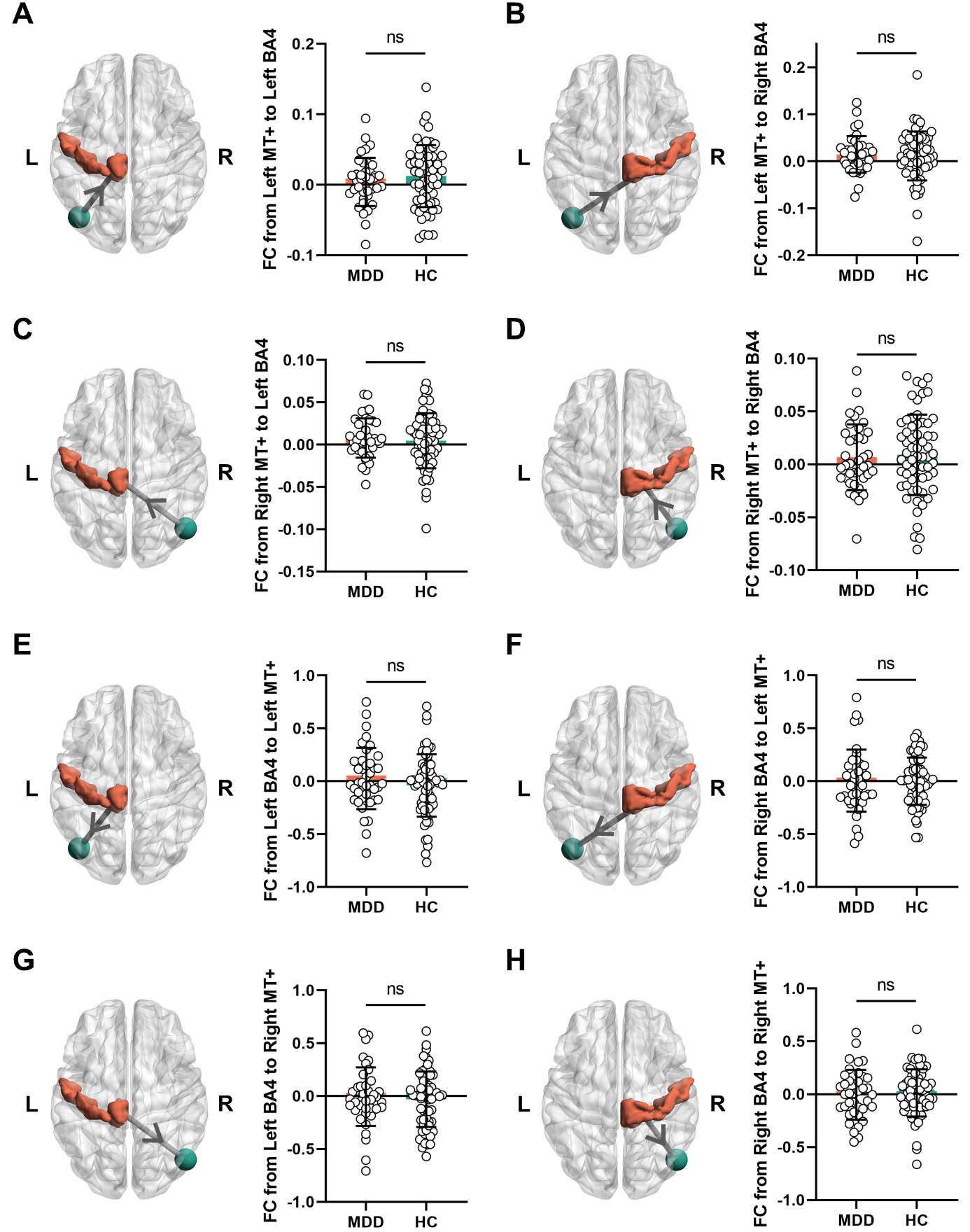


**Figure S7.** Comparison in effective FC between BA4 and MT+. There was no significant group difference in effective FC from MT+ to BA4 (A-D) and effective FC from BA4 to MT+ (E-H) between MDD and HC groups (Student’s *t* test). ns *P* > 0.05. L, left; R, right; FC, functional connectivity; BA, Brodmann Area; MT+, middle temporal visual cortex complex; MDD, major depressive disorder; HC, healthy control.


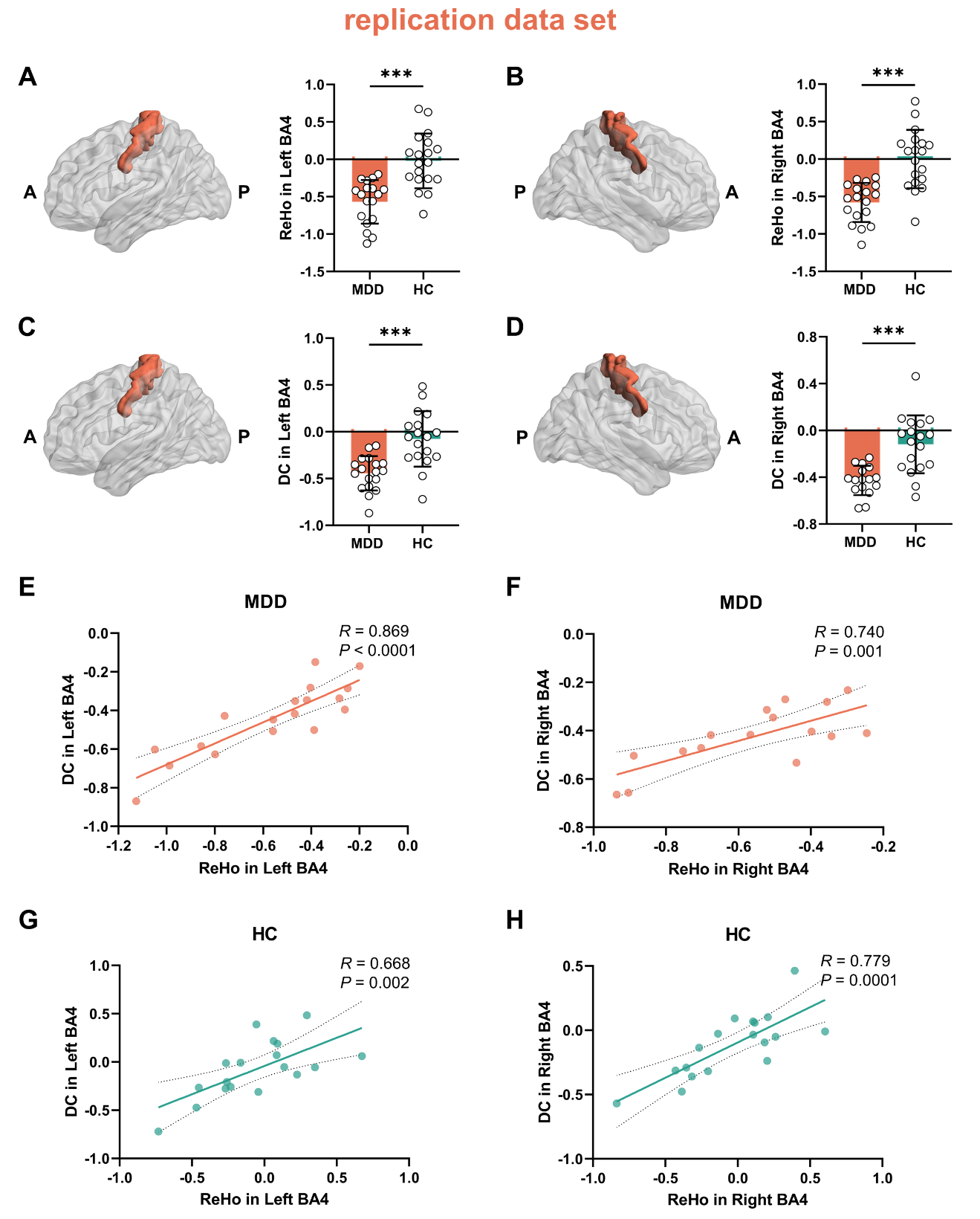


**Figure S8.** Comparison of ReHo and DC within BA4 between MDD and HC groups of the replication data set. MDD showed significantly reduced ReHo (A and B) and DC (C and D) in left and right BA4 compared with HC (Student’s *t* test). E-H) DC is related to ReHo in left and right BA4 in MDD and HC groups (Pearson correlation). *** *P_FDR_* < 0.001. A, anterior; P, posterior; ReHo, regional homogeneity; DC, degree centrality; BA, Brodmann Area; MDD, major depressive disorder; HC, healthy control.


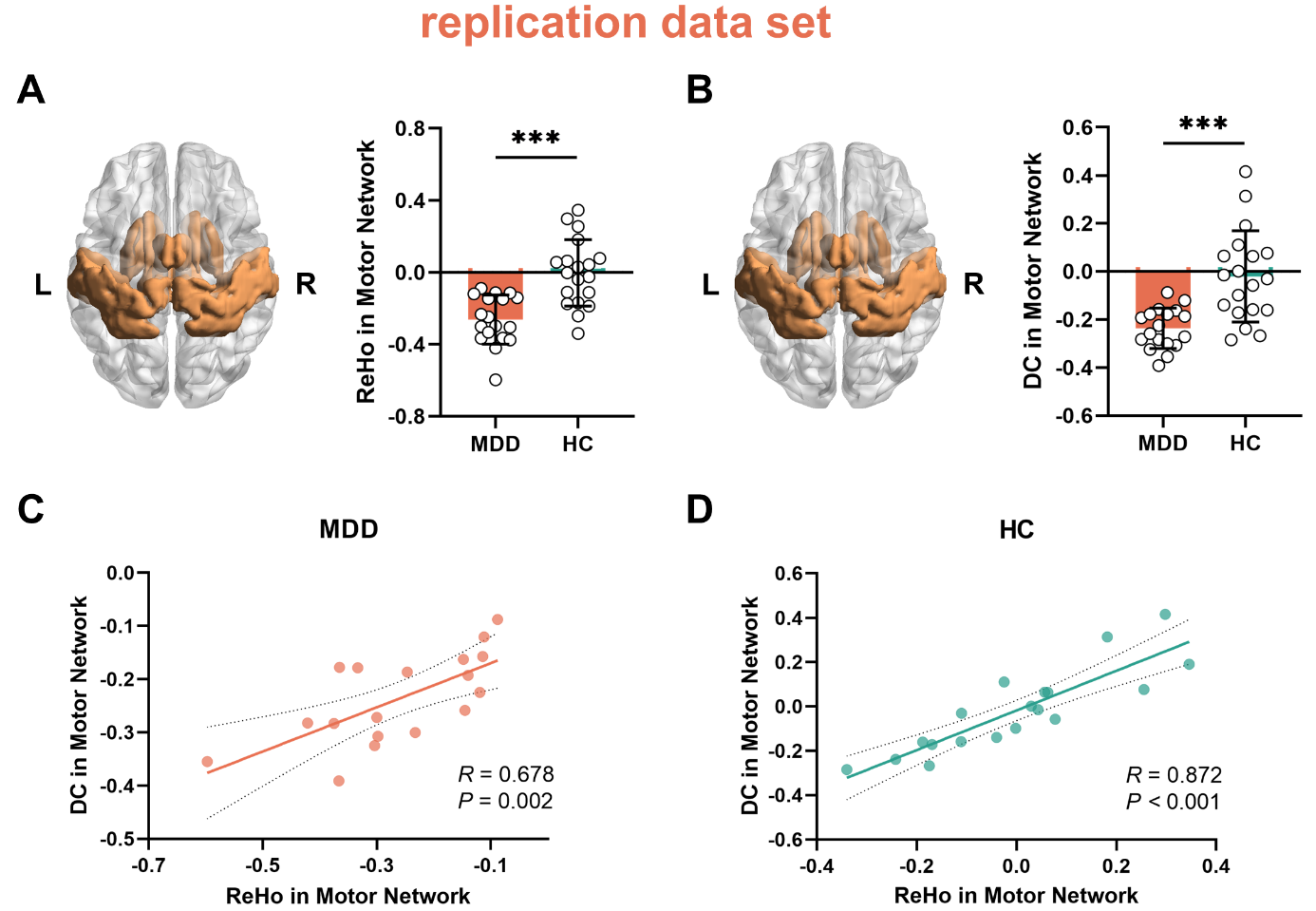


**Figure S9.** Comparison and relationships in ReHo and DC within motor network in replication data set. Reduced ReHo (A) and DC (B) in motor network in MDD group compared to HC group (Student’s *t* test). Correlation between ReHo and DC in motor network in MDD (C) and HC (D) groups (Pearson correlation). *** *P* < 0.001. L, left; R, right; MDD, major depressive disorder; HC, healthy control.


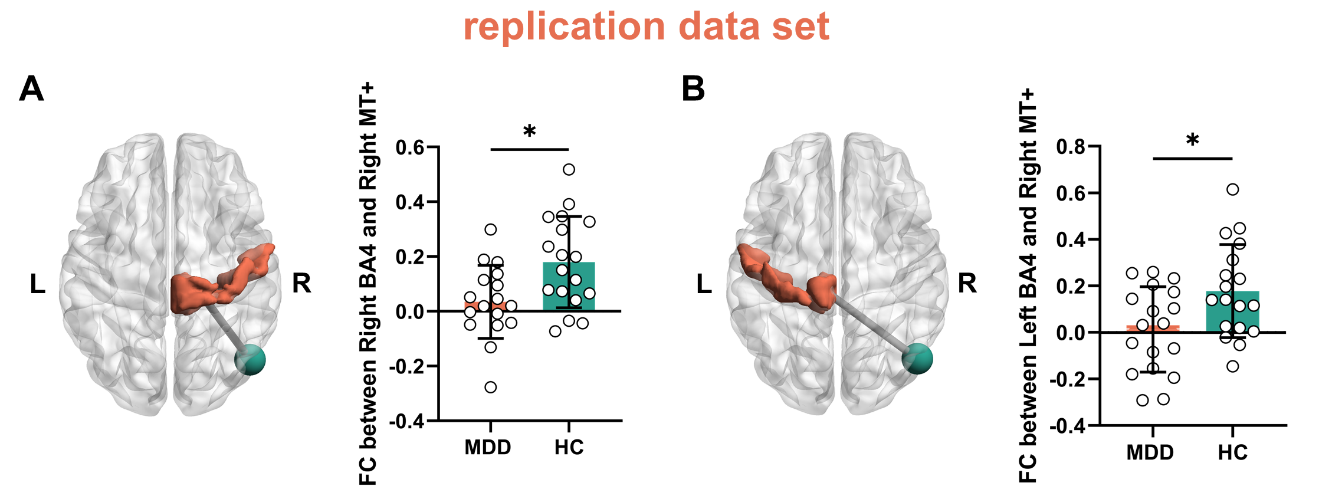


**Figure S10.** Comparison of the functional connectivity (FC) between BA4 and right MT+ between MDD and HC groups in replication data set. Reduction in FC between left (A) and right BA4 (B) and right MT+ (Student’s *t* test). * *P_FDR_* < 0.05. L, left; R, right; FC, functional connectivity; BA, Brodmann Area; MT+, middle temporal visual cortex complex; MDD, major depressive disorder; HC, healthy control.


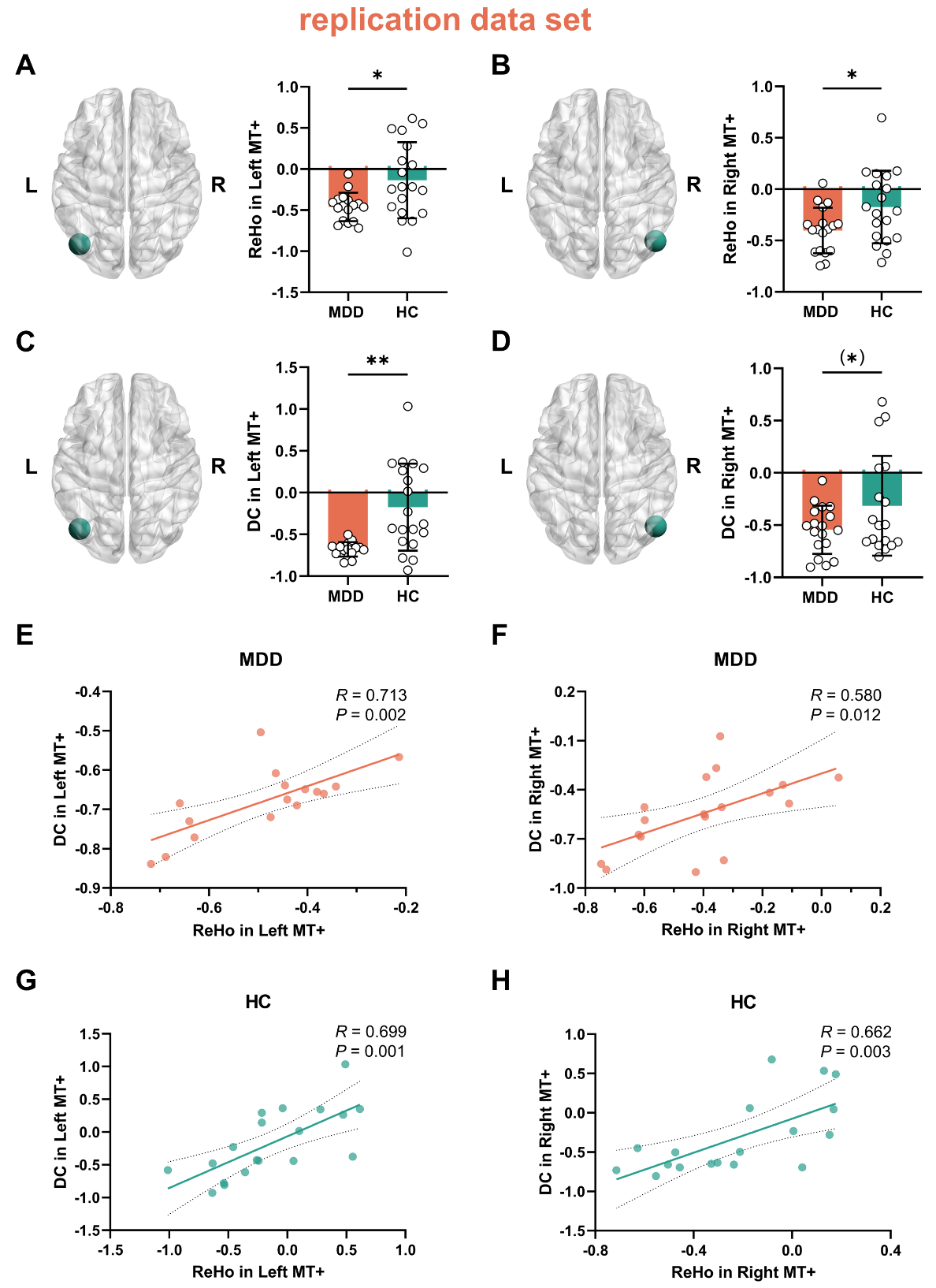


**Figure S11.** Group differences and relationships of DC and ReHo within MT+ in the replication data set. ReHo in left (A) and right MT+ (B) were significantly decreased in MDD group (Student’s *t* test). C) Reduction in DC within left MT+ in MDD patients compared to HC group (Student’s *t* test). D) DC in right MT+ was marginally decreased in MDD group (Student’s *t* test). E-H) Correlation between DC and ReHo within left and right MT+ in MDD and HC groups (Pearson correlation). (*) *P_FDR_* < 0.1, * *P_FDR_* < 0.05, ** *P_FDR_* < 0.01. L, left; R, right; MT+, middle temporal visual cortex complex; ReHo, regional homogeneity; DC, degree centrality; MDD, major depressive disorder; HC, healthy control.


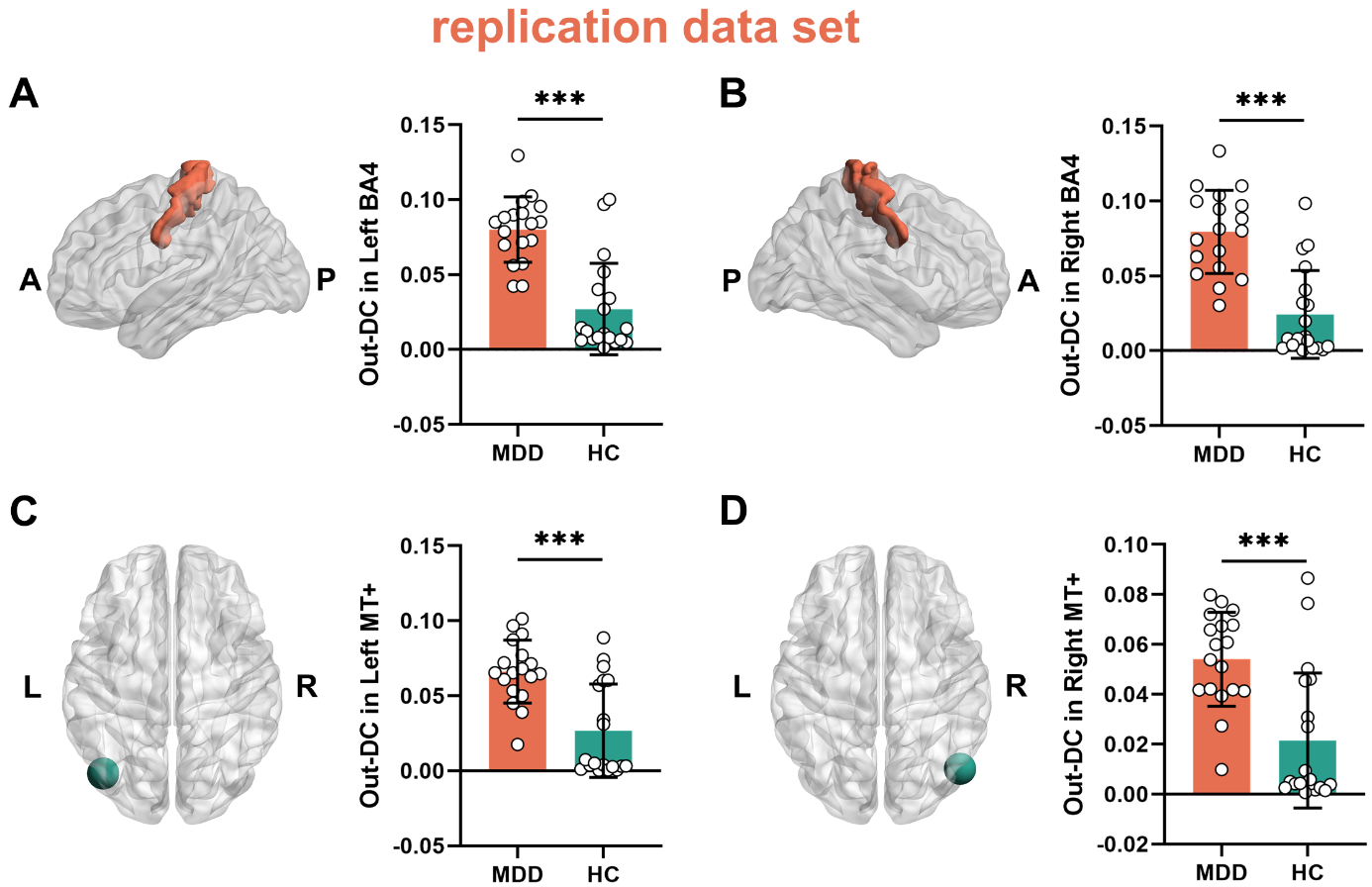


**Figure S12.** Comparison in out-DC within BA4 and MT+ between MDD and HC groups in replication data set. A) Out-DC was significant increased in left BA4 in MDD group (Student’s *t* test). B) Increased out-DC in right BA4 in MDD group (Student’s *t* test). Out-DC was significantly increased in left MT+ (C) and right MT+ (D) (Student’s *t* test). Out-DC, DC in output direction. *** *P* < 0.001. A, anterior; P, posterior; L, left; R, right; BA, Brodmann Area; MT+, middle temporal visual cortex complex; MDD, major depressive disorder; HC, healthy control.


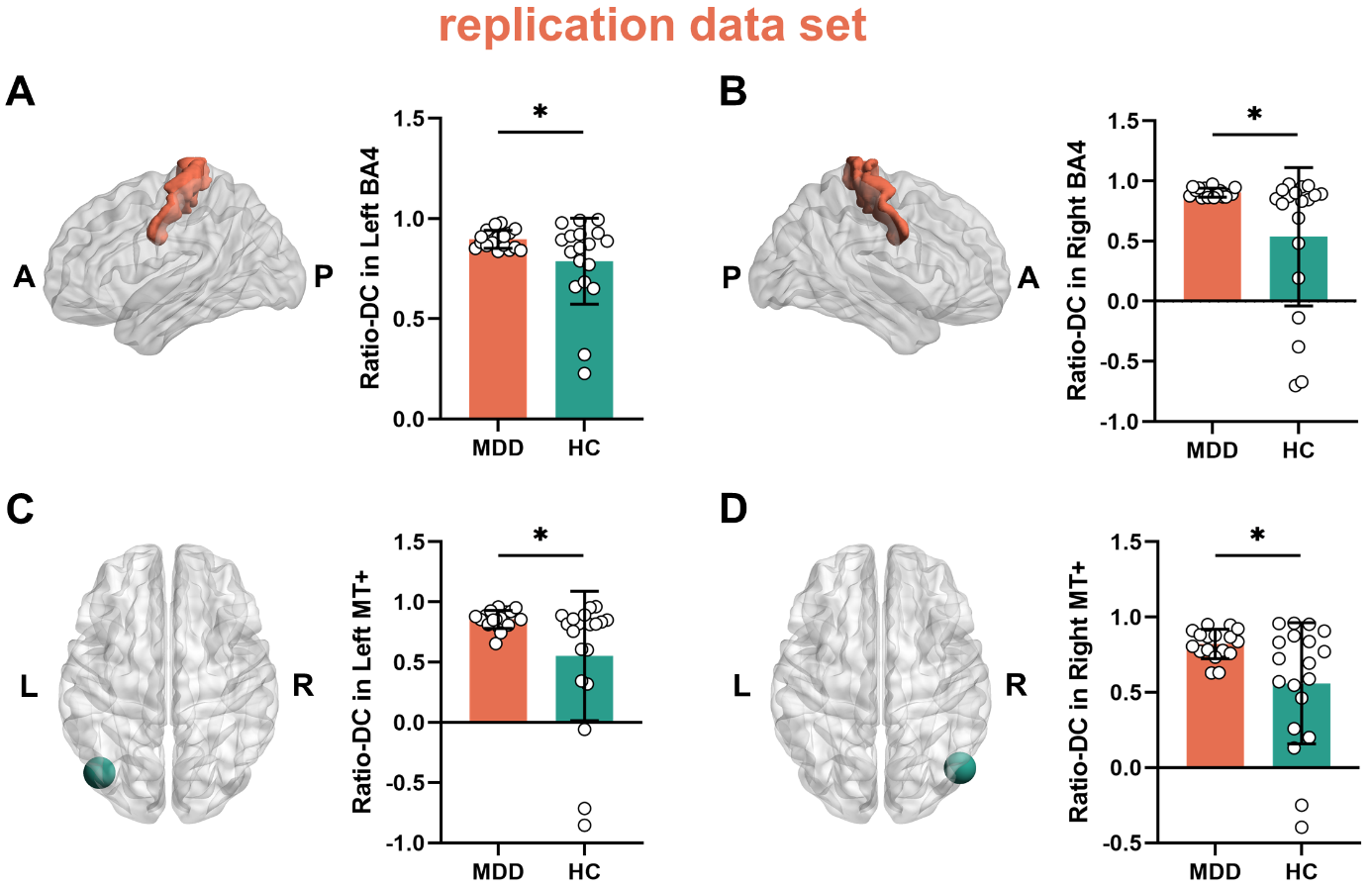


**Figure S13.** Group differences in Ratio-DC within BA4 and MT+ in replication data set. A) Compared to HC group, ratio-DC was significant increased in left BA4 in MDD group (Student’s *t* test). B) Increased ratio-DC in right BA4 in MDD group (Student’s *t* test). C) Ratio-DC was significantly increased in left MT+ in MDD group (Student’s *t* test). D) Increased ratio-DC in right MT+ in MDD group (Student’s *t* test). * *P* < 0.05. A, anterior; P, posterior; L, left; R, right; BA, Brodmann Area; MT+, middle temporal visual cortex complex; MDD, major depressive disorder; HC, healthy control.


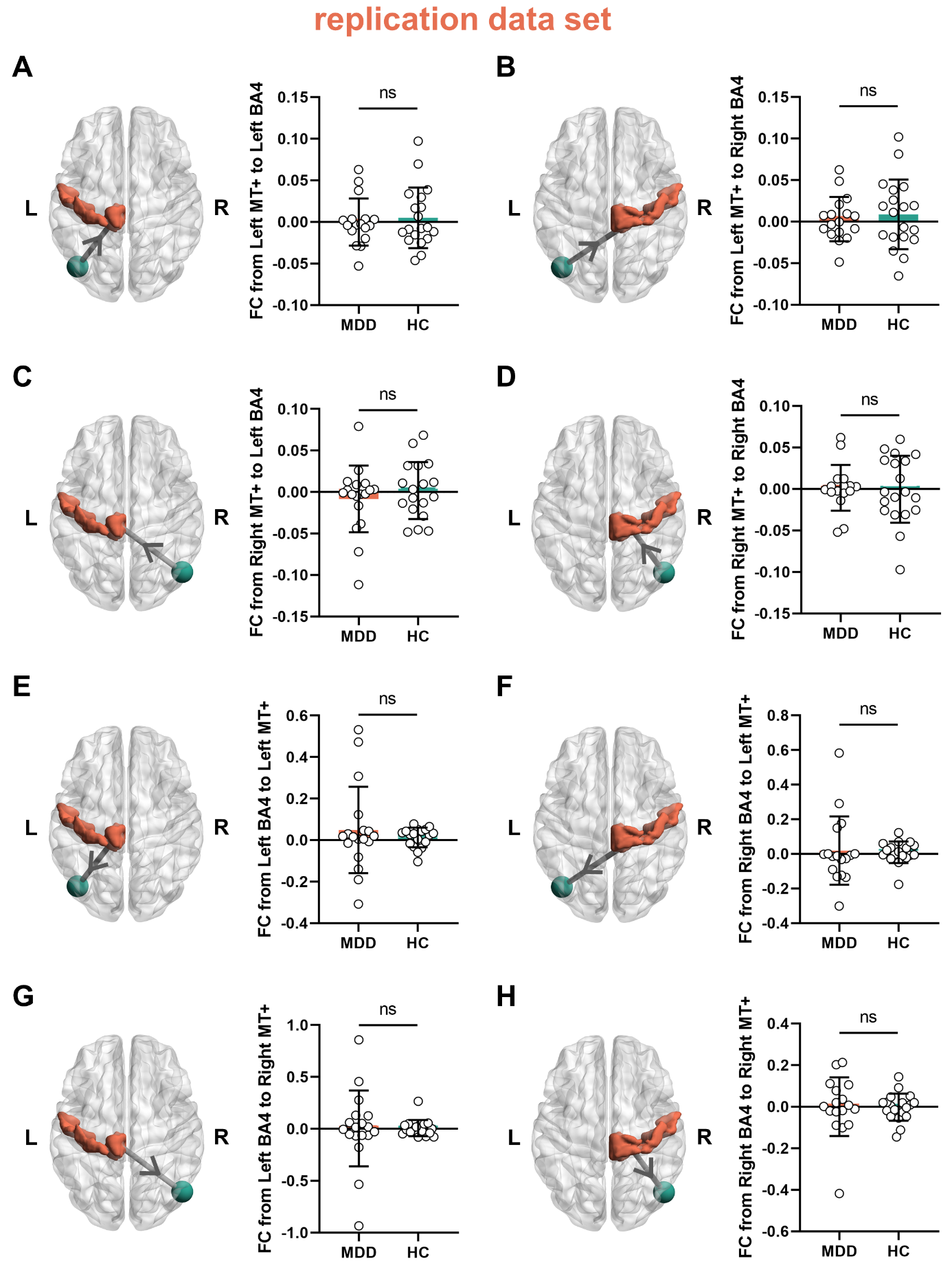


**Figure S14.** Group differences in effective FC between BA4 and MT+ in replication data set. There was no significant group difference in effective FC from MT+ to BA4 (A-D) and effective FC from BA4 to MT+ (E-H) between MDD and HC groups (Student’s *t* test). ns *P* > 0.05. L, left; R, right; FC, functional connectivity; BA, Brodmann Area; MT+, middle temporal visual cortex complex; MDD, major depressive disorder; HC, healthy control.


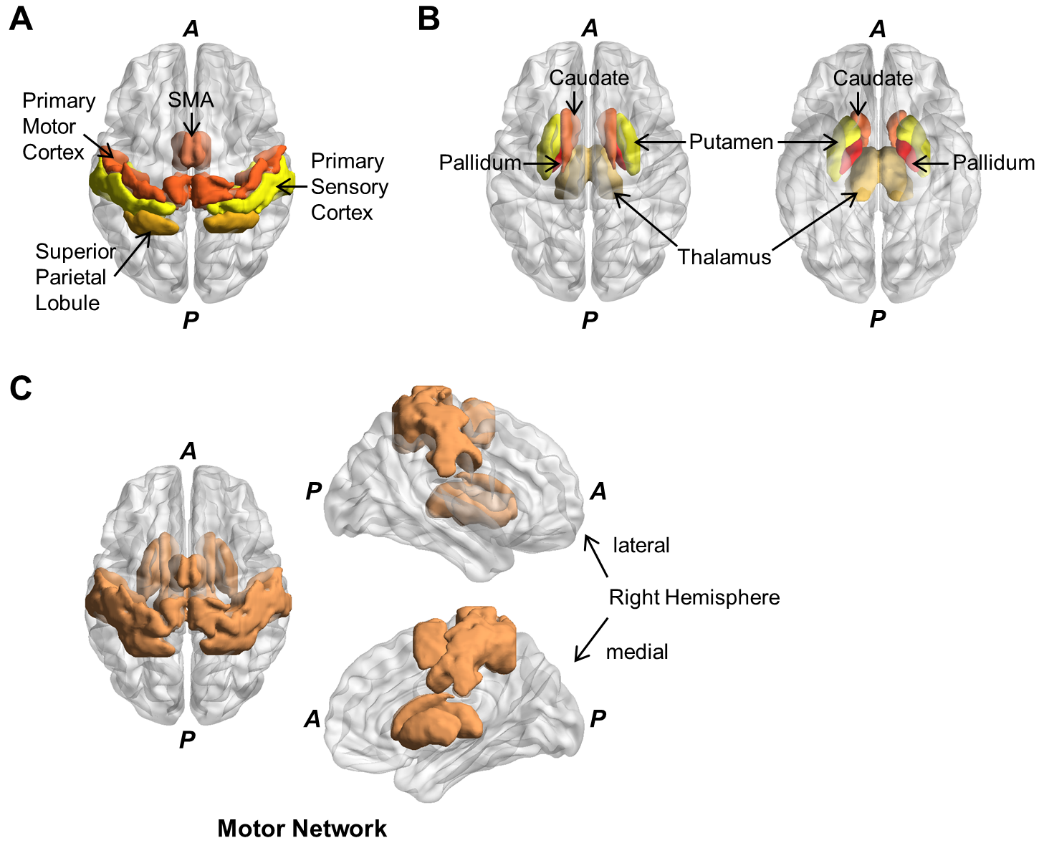


**Figure S15.** Schematic diagram of the motor network. A) Cortical motor regions included primary motor cortex (Brodmann Area 4), primary sensory cortex, superior parietal lobule and SMA. B) Subcortical motor regions included thalamus, caudate, putamen and pallidum. C) Motor network mask consisted of above cortical and subcortical motor regions. *A*, anterior; *P*, posterior; SMA, supplementary motor area.
